# A Single‐Pot Template Reaction Towards a Manganese‐Based *T*_1_ Contrast Agent

**DOI:** 10.1002/ange.202100885

**Published:** 2021-04-01

**Authors:** Sellamuthu Anbu, Sabrina H. L. Hoffmann, Fabio Carniato, Lawrence Kenning, Thomas W. Price, Timothy J. Prior, Mauro Botta, Andre F. Martins, Graeme J. Stasiuk

**Affiliations:** ^1^ Department of Biomedical Sciences University of Hull Cottingham Road Hull HU6 7RX UK; ^2^ Department of Chemistry University of Hull Cottingham Road Hull HU6 7RX UK; ^3^ Werner Siemens Imaging Center Department of Preclinical Imaging and Radiopharmacy Eberhard Karls University Tübingen, Röntgenweg 13/1 72076 Tübingen Germany; ^4^ Dipartimento di Scienze e InnovazioneTecnologica Università del Piemonte Orientale “A. Avogadro” Viale Teresa Michel 11 15121 Alessandria Italy; ^5^ MRI centre Hull Royal Infirmary Hospital NHS Trust Anlaby Road Hull HU3 2JZ UK; ^6^ Department of Imaging Chemistry and Biology School of Biomedical Engineering and Imaging Sciences King's College London Fourth Floor Lambeth Wing St Thomas' Hospital London SE1 7EH UK; ^7^ Cluster of Excellence iFIT (EXC 2180) “Image-Guided and Functionally Instructed Tumor Therapies” University of Tuebingen Germany

**Keywords:** bishydrated MnCA, blood pool agents, in vivo MRI, Mn-based contrast agents, template synthesis

## Abstract

Manganese‐based contrast agents (MnCAs) have emerged as suitable alternatives to gadolinium‐based contrast agents (GdCAs). However, due to their kinetic lability and laborious synthetic procedures, only a few MnCAs have found clinical MRI application. In this work, we have employed a highly innovative single‐pot template synthetic strategy to develop a MnCA, **MnL^Me^**, and studied the most important physicochemical properties in vitro. **MnL^Me^** displays optimized *r*_1_ relaxivities at both medium (20 and 64 MHz) and high magnetic fields (300 and 400 MHz) and an enhanced *r*_1_^b^=21.1 mM^−1^ s^−1^ (20 MHz, 298 K, pH 7.4) upon binding to BSA (*K*_a_=4.2×10^3^ M^−1^). In vivo studies show that **MnL^Me^** is cleared intact into the bladder through renal excretion and has a prolonged blood half‐life compared to the commercial GdCA Magnevist. **MnL^Me^** shows great promise as a novel MRI contrast agent.

## Graphical abstract

We present a single‐pot template synthesis strategy for a manganese‐based MRI contrast agent, **MnL^Me^**. **MnL^Me^** is highly inert toward zinc‐transmetallation and displays enhanced *T*_1_ relaxivity upon non‐covalent interaction with serum albumin. In vivo studies show higher contrast enhancement in the liver and longer blood half‐life than that of the commercially available contrast agent Magnevist. **MnL^Me^** shows great potential for use as a blood pool agent.

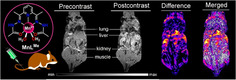

## Introduction

Magnetic resonance imaging (MRI) is a powerful non‐invasive medical imaging technique that produces high‐resolution 3D images of anatomy and the physiological function.^[1]^ MRI monitors the difference between longitudinal (*T*_1_) and transverse (*T*_2_) relaxation times of water protons in the body. Different tissues in the body present different *T*_1_ and *T*_2_ values due to the differential water content and chemical surroundings of the water protons, which allows in some cases to differentiate healthy from diseased tissues. However, the inherent contrast offered by the water relaxation times (*T*_1_ and *T*_2_) of normal and abnormal tissues is often subtle, which limits the accuracy of detection.^[1a]^ The contrast of specific organs, tissues, and disease progression, is enhanced when contrast agents (CAs) are applied to shorten the relaxation rate (*R*_1,2_=1/*T*_1/2_) of water protons within these tissues.

Gadolinium‐based MRI contrast agents (GdCAs) have been applied since 1988 and used in clinics due to their kinetic inertness, fast clearance, and favorable hepatic/renal/hepatobiliary elimination.^[2]^ Nevertheless, free Gd^3+^ ions are toxic in biological systems due to the similarity in ionic radius (0.94 Å) with that of the Ca^2+^ ion (0.99 Å).^[3]^ Recently, due to the weaker kinetic stability of acyclic GdCAs particularly when applied in vivo, a red flag has been raised for most of the clinical GdCAs due to aggravated nephrogenic systemic fibrosis (NSF)[^4^, ^5^] and the detection of Gd^3+^ deposition in the brain upon recurrent injection of GdCAs.^[6]^ Due to these concerns, the European Medicines Agency (EMA) has suspended the marketing authorizations of the four acyclic GdCAs.[^7^, ^8^] The US—Food and Drug Administration (FDA) also advised against the use of linear acyclic GdCAs in patients with compromised renal function.[^7^, ^9^] To overcome these hurdles, it is essential to develop and implement Gd^3+^‐free MR contrast agents to clinical routine. Manganese‐based contrast agents (MnCAs) have been introduced as safer alternatives^[10]^ to GdCAs. Mn^2+^ has five unpaired *d* electrons, long electronic relaxation time, and rapid water exchange. These properties are ideal for CAs capable of enhancing *T*_1_ contrast.^[11]^ Manganese is a biogenic and vital metal found in most tissues (mean serum concentration of 0.5–1.2 μg L^−1^). It is required for bone development, neuronal health and, other physiological and cellular (mitochondrial) functions.^[12]^ It plays an essential role in amino acid, lipid, protein, and carbohydrate metabolism.^[12a]^ It is also involved in glycogen storage in the liver, protein digestion, and the synthesis of cholesterol and fatty acids.^[13]^ Despite the increased interest in MnCAs development, no MnCAs are clinically available.^[14]^ Therefore, there is significant interest in the development of next‐generation MnCAs, with 1) high thermodynamic stability, 2) kinetic inertness under physiological conditions, and 3) fast in vivo clearance. A major challenge in developing MnCAs is that Mn^2+^ shows a limited crystal field stabilization‐lower thermodynamic stability‐by most ligands compared to the other transition metals from the Irving‐Williams series.[^15^, ^16^] It is thus of critical importance to design MnCAs with superior kinetic inertness. As demonstrated for lanthanide‐based complexes, kinetic inertness is crucial when applying safe CAs in vivo.^[17]^

The MRI contrast efficiency of CAs, the relaxivity (*r*_1_), is proportional to the number of water molecules present in its first coordination sphere.[^18^, ^19^, ^20^, ^21^, ^22^] Despite the advantageous capabilities of producing brighter MR contrast, only a few bishydrated MnCAs have been reported so far, and none showed favorable kinetic inertness for in vivo applications.[^16^, ^23^] In particular, pyridyl‐based pentadentate ligands with N_5_ or N_3_O_2_ donors have seen interest due to the formation of a stable pentacoordinate planar coordination structure with Mn^2+^.^[24]^ This structure provides an additional feature for coordinating two additional axial water molecules or counter anions.^[25]^ Another critical parameter for the efficiency of contrast agents is a slow rate of molecular reorientation defined as the rotational correlation times (*τ*_R_). This can be achieved by developing macromolecular contrast agents (i.e., proteins, functionalized gold nanoparticles, quantum dots, dendrimers, nanotubes, liposomes)[^26^, ^27^] by conjugating the paramagnetic metal complex into a slowly tumbling macromolecule,^[28]^ or incorporating functional groups that are capable of interacting non‐covalently with macromolecules such as blood plasma proteins.^[29]^ The latter received great attention as they render opportunities to design and synthesize contrast agents with specific groups that target macromolecules to prolong their lifetime in the bloodstream.[^30^, ^31^] Thus there is need to develop new MnCAs bearing hydrophobic groups, to facilitate the binding to human serum albumin (HSA) and improve the relaxometric properties and pharmacokinetics, which result in optimal blood pool agents.

Herein, we report a new, economic, and straightforward one‐pot synthesis of a Schiff‐base type diacetylpyridyl carbohydrazide‐based Mn^2+^ complex (**MnL^Me^**) as a *T*_1_ contrast agent. **MnL^Me^** showed kinetic inertness to zinc‐transmetallation and displayed enhanced *T*_1_ relaxivity upon non‐covalent binding with serum albumin. The kinetic inertness of the acyclic Mn^2+^ complex (**MnL^Me^**) has been examined concerning time course relaxivity, zinc‐transmetallation, spectral absorption titration, in vivo excretion stability and NMRD experiments. In vivo, MRI was also conducted in healthy mice to assess *T*_1_ enhancement in tissues, biodistribution, and elimination pathways of **MnL^Me^**.

## Results and Discussion

### Design, Syntheses, and Structural Analyses

**Scheme 1:**
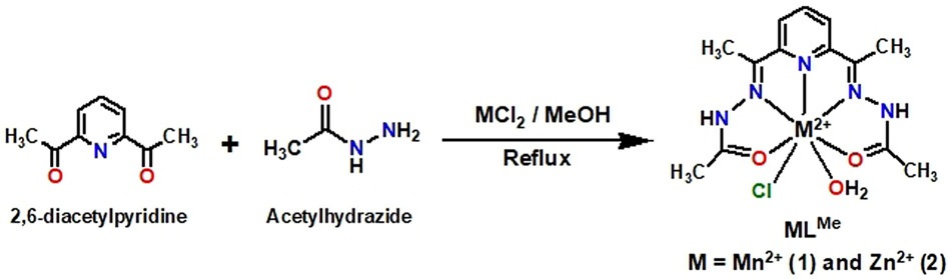
Synthesis of complexes **MnL^Me^** and **ZnL^Me^**.

We have synthesised a Schiff‐base type (2,6‐diacetylpyridine bisacetylhydrazone) manganese complex in a facile single‐pot template method. The mononuclear Mn^2+^ complex **MnL^Me^** was synthesised by refluxing 1:2:1 molar mixture of 2,6‐diacetylpyridine, acetyl hydrazine and MnCl_2_ in methanol (Scheme 1). The isolated yield of the **MnL^Me^** was found to be 91 %, which is higher than the yields of other derived heptacoordinated Mn^2+^ complexes.[^25b^, ^32^, ^33^] To explore the structure of the ligand **L^Me^** by NMR, we synthesised the diamagnetic Zn^2+^ analogue **ZnL^Me^** along with **MnL^Me^**; both complexes were fully characterized by elemental analyses, conventional spectroscopy, and X‐ray crystallographic techniques. The ^1^H‐NMR of **ZnL^Me^** in D_2_O (Figure S1) shows a single set of aromatic signals (*δ*=8.2 to 8.0 ppm), and two resonances at *δ*=2.23 to 2.44 ppm corresponding to the aliphatic methyl protons. The ^13^C‐NMR spectrum of **ZnL^Me^** (Figure S2) is consistent with the proposed structure. It displays seven expected resonances, 12.6 ppm, and 19.7 ppm for the CH_3_ nuclei, a peak at 124.5 ppm for the azomethines (C=N), three aromatic signals between 143 ppm and 148 ppm and a single resonance at 173 ppm corresponds to the carbonyl groups.

The complexes (**MnL^Me^** and **ZnL^Me^**) are highly soluble (≈96 and 84 mg mL^−1^, respectively) in 50 mM HEPES buffer (pH 7.3). They display two intense absorption bands at 213 and 275 nm, attributed to the intra‐ligand π‐π* and n‐π* transitions within the coordinated pyridyl ring and electron localized carbohydrazones (Figure S3). The MS (ESI^+^) analysis of **MnL^Me^** and **ZnL^Me^** in methanol (Figures S4, S5) confirms 1:1 stoichiometry between chelate and M^2+^ ion (M=Mn and Zn), where the spectral profiles exhibit peaks at *m*/*z* of 365.045 & 329.068 and 374.18 & 339.14 corresponding to the expected ([*M*(L^Me^)(Cl^−^)]^+^) and ([*M*(L^Me^−H^+^)]^+^) species, respectively. Overall, the spectroscopic and analytical data are consistent with the proposed structural formula of the complexes.

### Crystal Structures of MnL^Me^ and ZnL^Me^

**Figure 1:**
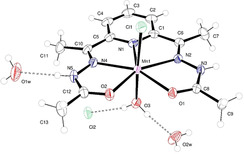
(Top) ORTEP diagram of the asymmetric unit of **MnL^Me^** with atoms drawn as 50 % thermal ellipsoids. The dashed lines show classical hydrogen bonds within the asymmetric unit.^[53]^

The Mn^2+^ complex **MnL^Me^** crystallizes in monoclinic space group *P*2_1_/*c* with a single molecule in the asymmetric unit (*Z*=4). The ORTEP diagram for **MnL^Me^** is presented in Figure 1. The Mn^2+^ ion is formally 7‐coordinate in a pentagonal bipyramidal geometry. The equatorial plane is composed of the N_3_O_2_ coordination environment of the PyCOHz ligand; the Mn‐N distances lie in the range 2.2872(18) to 2.979(10) Å and the Mn‐O1 and Mn‐O2 distances are 2.2446(16) and 2.2418(16) Å, respectively. The axial ligands, chloride (Mn‐Cl1=2.499(8) Å), and water (Mn‐O3=2.173(2) Å) show little deviation from linearity (∡Cl1‐Mn‐O4=177.17(5)°). Bite angles between each of the equatorial N_3_O_2_ donors and axial Cl1 and H_2_O are >90° and <90°, respectively, which reveals that the Mn atom is lying slightly away (0.1103(10) Å) from the mean basal plane towards the axial Cl1 atom. The relatively long Mn‐Cl1 bond distance indicates that the Cl1 is weakly coordinated to the Mn^2+^ and likely to be replaced by a water molecule in aqueous solution.[^22b^, ^34^] The E1‐Mn1‐E2 angles, where E1 and E2 denote equatorial donor atoms (N_3_O_2_) of the PyCOHz plane, fall within the range 68.08–70.24° which is very close to the 72° for ideal pentagonal bipyramidal coordination.

The analogous Zn^2+^ complex **ZnL^Me^** is isostructural (Figure S6) with the Mn^2+^ complex **MnL^Me^** and the Zn^2+^ ion lying very close (0.078(2) Å) to the basal N_3_O_2_ plane. Within the structures of each complex, there are intermolecular hydrogen bonds between the complex (water, chloride) and further unbound water and chloride. The extensive 3D hydrogen‐bonding network in the solid state for **MnL^Me^** is described and illustrated in the supporting information (Figure S7). Crystallographic data and selected bond distances and angles of both the complexes (**MnL^Me^** and **ZnL^Me^**) are summarized in Tables S1–S10. Also, it is worth noting that the crystal structures of **MnL^Me^** and **ZnL^Me^** are similar to those of the analogous complexes [**M**(DAPSC)(Cl)(H_2_O)]Cl⋅2 H_2_O for **M**=Mn, Zn (where DAPSC=2,6‐diacetylpyridine bis(semicarbazone).^[24]^ These differ only in the ligand (the pair of terminal methyl groups in **L^Me^** are NH_2_ in DAPSC) and this is reflected in the crystal structure (See SI).

### Relaxometry Studies

The longitudinal proton relaxivity *r*_1_ is the measure of the contrast capability of the agent (per unit of agent per second). The longitudinal relaxation times (*T*_1_) of **MnL^Me^** at different concentrations were measured at both 400 MHz (9.4 Tesla) and 64 MHz (1.5 Tesla) at 298 K. The relaxivity values *r*_1_ of **MnL^Me^** at 9.4 and 1.5 T were determined as 4.88±0.4 and 5.2 mM^−1^ s^−1^, respectively, from the slope of corresponding plots of 1/*T*_1_ vs. [**MnL^Me^**] (Figure S8). To corroborate the MR capability of **MnL^Me^**, *T*_1_‐weighted phantom MR images of the complex at different concentrations (0.1–0.5 mM and 1–5 mM) and Magnevist® (clinically available acyclic GdCA, [Gd(DTPA)(H_2_O)]^2−^) were evaluated using 7 T preclinical and 1.5 T clinical MRI systems, respectively (Figures 3 and S8). The relaxivity of **MnL^Me^** is attributed to the presence of two coordinated water molecules (it is postulated that the bound Cl anion in the crystal structure is replaced in situ). **MnL^Me^** displays enhanced relaxivity in comparison to the reported monohydrated Mn^2+^ complexes and comparable *r*_1_ to the bishydrated.[^23^, ^25^] **MnL^Me^** also shows a higher *r*_1_ value in comparison to that of Gd‐DTPA (3.6 mM^−1^ s^−1^ at 1.5 T and 7 T 298 K); this is reflected in the phantom image of Gd‐DTPA at 1 mM concentration (Figure S8). The pentacoordinate planar and rigid structure of the pyridyl carbohydrazone may also influence the electronic spin relaxation times of Mn^2+^ ion and shortening the residence lifetime of the inner‐sphere water molecules.[^22b^, ^22c^, ^23^]

The *r*_1_ value is inversely proportional to the length of the intramolecular metal‐water bond. So, the increased bond length between the metal ion and coordinated water molecule (M‐O_water_) suggests a more labile bond that could undergo rapid displacement with bulk solvent water.[^25b^, ^33a^, ^35^] However, in this complex, the bond length of Mn‐O_water_ is found to be 2.173(2) Å which is shorter than the Mn‐Cl distance (2.499(8) Å) suggesting the Cl can be replaced with water molecules from the bulk and form a bishydrated complex in the aqueous solution^[22b]^ leading to enhanced relaxivity compared to reported monohydrated Mn^2+^ complexes.[^19b^, ^20c^, ^28b^] Moreover, the distance between the water protons and Mn^2+^ ions (Mn⋅⋅⋅H_water_) was found to be 2.74 Å, which enables efficient dipole‐dipole coupling between the water protons and paramagnetic metal ion.[^29b^, ^36^]

The other important parameter for MnCAs is the kinetic inertness of the complex, crucial to in vivo application, which can be correlated with ligand transmetallation rates. We have performed a series of transmetallation experiments to ascertain the chelating and kinetic stability of **MnL^Me^**. Transmetallation of **MnL^Me^** was assessed and compared with the bench mark CAs including [Mn(EDTA)(H_2_O)]^2−^, [Mn(DTPA)]^3−^ and [Gd(DTPA)(H_2_O)]^2−^ in the presence of Zn^2+^ (5/25 molar equivalent) in 50 mM MES/HEPES at pH 6.0/7.4 (400 MHz, 9.4 T, 298 K). The initial water proton relaxation rates (*R*_1_=1/*T*_1_) of **MnL^Me^** (1 and 2 mM) were observed as 5.78±0.5 and 9.86 s^−1^, which increased slightly (*R*_1_≈6.15±0.4 and 10.69 s^−1^) upon addition of a 5 and 25 molar equivalent excess of Zn^2+^, respectively. This suggests a minor increase in molecular weight possible through the association of Zn^2+^ to the NH groups (*m*/*z*=439.31 **ZnL^Me^ + Zn**, Figure S5). Nevertheless, small and negligible fluctuations in *R*_1_ were observed for **MnL^Me^** with the excess (5 and 25 molar equivalent) of Zn^2+^ over the remaining 60 and 15 days of the experiment, respectively (Figures S9–S11). Notably, **MnL^Me^** displays remarkable (21, 50 and 9‐fold) kinetic inertness toward Mn^2+^ transmetallation than the [Mn(EDTA)(H_2_O)]^2−^, [Mn(DTPA)]^3−^ and [Gd(DTPA)(H_2_O)]^2−^, respectively, since their *R*_1_ values were substantially increased from 3.14±0.16, 1.84±0.2 and 5.28±0.4 s^−1^ to 7.35±0.4, 7.66±0.7 and 8.29±1.1 s^−1^, respectively, after addition of 25 molar equivalent of Zn^2+^ in 50 mM MES buffer at pH 6.0, 298 K.

**Figure 2:**
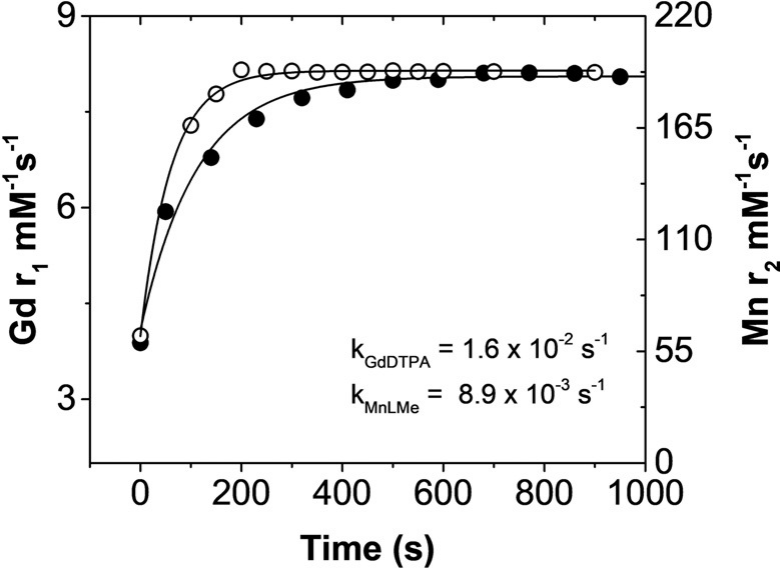
Transmetallation of **MnL^Me^** (•) and [Gd(DTPA)(H_2_O)]^2−^ (○) were separately incubated with 25 mol excess Zn^2+^ at pH 6.4 in 50 mM TRIS buffer. The *T*_1_ of water protons was measured using a Bruker NMR spectrometer (*B*_0_=14.1 T) over 900–950 seconds. The fitted lines reflect pseudo‐first order rate constants for dissociation of free Gd^3+^ from [Gd(DTPA)(H_2_O)]^2−^ and free Mn^2+^ from **MnL^Me^**.

In order to ascertain the Mn^2+^ and Gd^3+^ displacements precisely in **MnL^Me^** and [Gd(DTPA)(H_2_O)]^2−^, the transmetallation kinetics were recorded by measuring water *T*_2_ (Mn^2+^) and *T*_1_ (Gd^3+^), respectively, over 900–950 seconds (Figure 2). Interestingly, **MnL^Me^** displays higher inertness (pseudo‐first‐order, *k*=8.9×10^−3^ s^−1^) to Zn^2+^ (25 equivalent excess) than the [Gd(DTPA)(H_2_O)]^2−^ (*k*=1.6×10^−2^ s^−1^). Notably, the *k* value obtained for GdCA such as Gd(DTPA)(H_2_O)]^2−^ is similar to the value reported in the literature.^[28b]^ This indicates that **MnL^Me^** shows high inertness to Zn^2+^ even when compared to commercially available GdCAs.

The time‐dependent electronic absorption spectral study was also carried out to prove the chelating stability of the **MnL^Me^** in aqueous (50 mM HEPES buffer) solution at pH 7.4 over a week. The absorption spectrum of the **MnL^Me^** is characterized by two intense bands at 213 and 275 nm. (Figure S12). The calculated absorption intensity ratios (Abs_275 nm_/Abs_213 nm_) of **MnL^Me^** at pH 7.4 are relatively constant over a week suggesting that it's reasonably stable at physiological conditions.

To show the stability of **MnL^Me^** outside of physiological conditions the *r*_1_ of the **MnL^Me^** has been investigated at seven different pH conditions including 3.0, 5.2, 6.0, 7.3, 8.5, 9.3 and 10.0 (Figure S13) (9.4 T, 298 K). Interestingly, the *r*_1_ values between pH 3.0 and 7.3 are found to be 4.01±0.3, 4.43±0.2, 4.53±0.2, 4.88±0.2 mM^−1^ s^−1^, respectively. Only a slight decrease in *r*_1_ values observed at lower pH conditions suggest that it may be due to the protonation of imino nitrogen atoms that led to increased chelating stability rather than stepwise dissociation of **MnL^Me^** under acidic conditions. This was further supported by the high protonation constant value (*K* ^H^>13) for imino nitrogen containing compound with similar (CH_3_)C=N‐NH‐CO moiety.^[37]^ Notably, presence of methyl groups adjacent to imino nitrogen atoms facilitate to preserve the hydrogen bonding that stabilize the molecule at acidic conditions. However, the *r*_1_ values at pH 8.5 and 9.6 were observed as 4.56±0.3 and 3.64±0.2 mM^−1^ s^−1^, respectively. A gradual dropping of the *r*_1_ values at pH>8.5 indicates that it forms monohydrate/hydroxo complex ([MnL^Me^(H_2_O)(OH)]^+^) under basic conditions. We also observed yellow precipitation accompanied dropping of *r*_1_ value (0.01 mM^−1^ s^−1^) at pH 10.0. This is likely due to the formation of insoluble bishydroxo complex.

The chelating stability of **MnL^Me^** with and without the presence of challenging chelating agents (EDTA and DTPA) was further analyzed by LC‐MS method (Figure S14) using CH_3_CN/NH_4_OAc buffer gradient (0–5 % CH_3_CN over 10 min) at pH 7.0, 298 K. Notably, there was no considerable change (retention time of **MnL^Me^** at 8.7 min) even after incubating (30 min) with 25 molar equivalent excess of DTPA or EDTA.

The data indicates that the Schiff‐base type **MnL^Me^** is highly inert to trans‐chelation even under high‐stress conditions and suggest that the thermodynamic stability of **MnL^Me^** is comparably higher than the benchmark Mn‐complexes ([Mn(EDTA)(H_2_O)]^2−^ and [Mn(DTPA)]^3−^) with reported pM values >7.7 (pM=−log[Mn^II^free]).^[28b]^ Furthermore, we have accessed the in vivo stability of **MnL^Me^** after injection of a clinical dose of **MnL^Me^** in a healthy mouse. Urine samples were collected, analyzed by LC‐MS, and quantified by the Evan method at 14.1 T (Figure S15). At 40 min post intravenous **MnL^Me^** (0.1 mmol kg^−1^ bodyweight, 2.5×10^−6^ M) injection, 24.24 % of the applied **MnL^Me^** was excreted into the bladder in an intact form. This further confirms that **MnL^Me^** is being excreted intact form, with no signs of transmetallation or trans‐chelation by other biogenic metals or biomolecules, respectively, suggesting superior thermodynamic stability and kinetic inertness in vivo.

### NMRD and ^17^O‐NMR Analyses

To gain more insight in the relaxivity values observed for **MnL^Me^**, we have recorded ^1^H Nuclear Magnetic Relaxation Dispersion (NMRD) profiles in aqueous solution at 283, 298 and 310 K in the proton Larmor frequency range 0.01–120 MHz, corresponding to magnetic field strengths varying between 2.34×10^−4^ and 2.81 T (Figure S16). The temperature‐dependent ^1^H‐NMRD profiles of **MnL^Me^** are typical of monomeric, low molecular weight complexes, featuring a low‐field plateau (0.01–1 MHz) followed by a dispersion about 4 MHz and another region of nearly constant relaxivity values above 20 MHz.^[38]^ In these cases, the *r*_1_ is limited to a large extent by the rotational correlation time, as typically observed in clinically used GdCAs.^[39]^ Support for this conclusion comes from the temperature dependency of *r*_1_ that decreases with increasing temperature across the entire frequency range investigated. Then, we can infer that **MnL^Me^** is in the fast‐water exchange regime[^38b^, ^39^] as further confirmed by the quasi‐exponential decrease of the relaxivity with the increase in temperature in the range 285–325 K (22 MHz; Figure S17). A least‐squares fit of the profiles of **MnL^Me^** was carried out according to the established theory of paramagnetic relaxation expressed by the Solomon‐Bloembergen‐Morgan and Freed's equations for the inner‐ (IS) and outer sphere (OS) proton relaxation mechanisms, respectively.[^25a^, ^25c^]

The hydration state of Mn^2+^ was confirmed by applying the empiric equation recently suggested by Peters and Geraldes.^[40]^ Using this approach, we obtained *q*=2.3±0.4, which is in excellent agreement with the determined *q*=2 value. Finally, the diffusion coefficient (^298^ *D*_MnH_) and its activation energy (*E*_DMnH_) were fixed to the values for the self‐diffusion of water molecules in pure water.^[41]^

**Figure 3:**
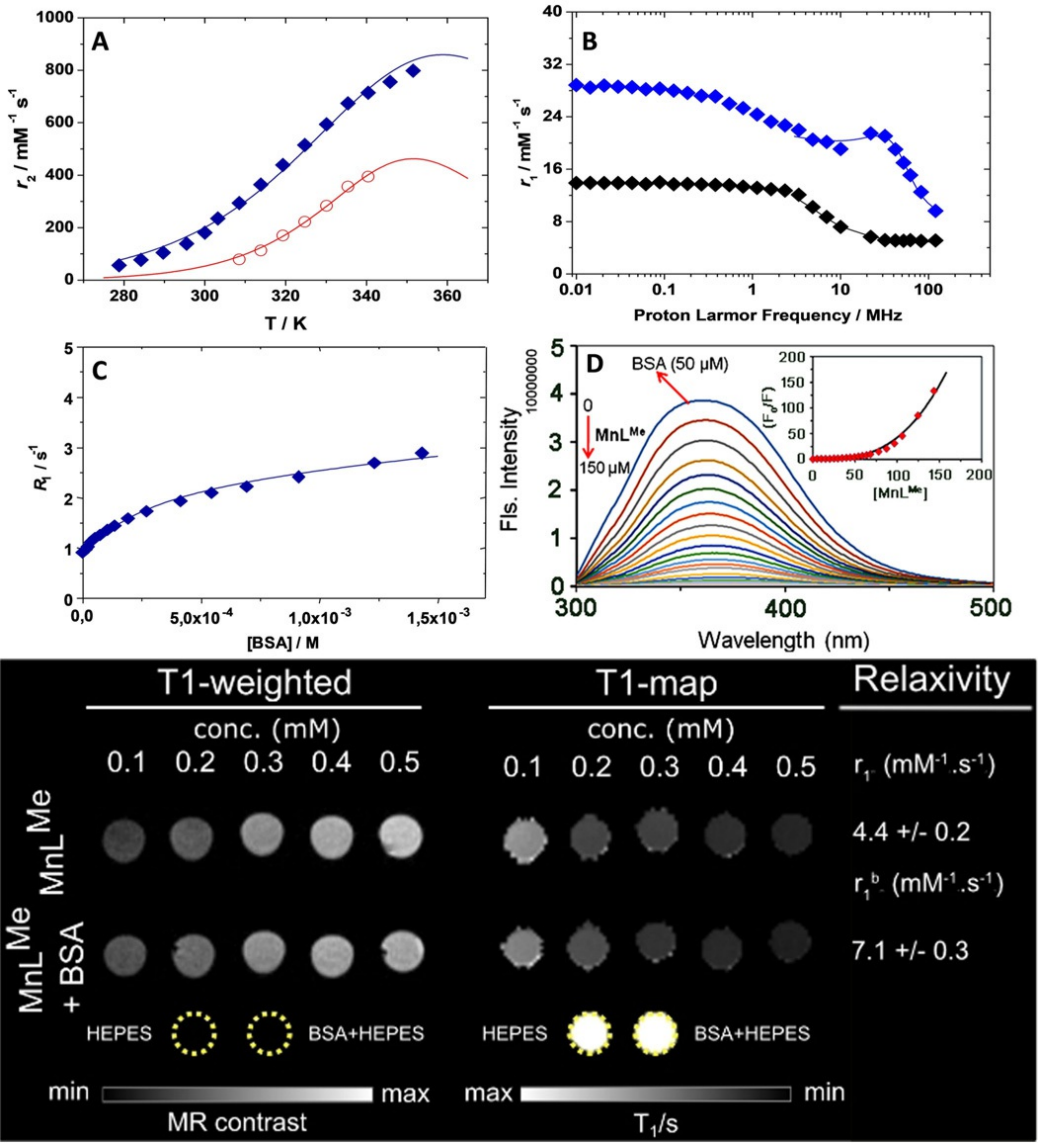
A) Temperature dependent^17^O NMR transverse relaxation rates of **MnL^Me^** free (blue diamonds) and bound to BSA (red circles). B) 1/*T*_1_ NMRD profiles measured at 298 K of **MnL^Me^** free (black) and bound to BSA (blue). C) Water proton relaxation rate (*R*_1_^obs^) as a function of BSA concentration for a 0.1 mM solution of **MnL^Me^** in 50 mM HEPES buffer at pH 7.4, 298 K, and 32 MHz. D) Emission spectral trace of 50 μM BSA protein in 50 mM HEPES buffer (pH 7.4, 298 K) with increasing quantity of complex **MnL^Me^** (0–150 μM, *λ*_ex_=280 nm); and inset Stern–Volmer plot of emission intensity *F*_0_/*F* vs. [**MnL^Me^**]. E) *T*_1_‐weighted imaging and *T*_1_‐maps of 0.1–0.5 mM **MnL^Me^** solutions (top row) and **MnL^Me^** at the same concentrations with 0.6 mM BSA (bottom row). Samples were prepared in 50 mM HEPES buffer at pH 7.0, and imaging was performed at 7.0 T and 310 K.

The temperature dependence of ^17^O NMR transverse relaxation rates allow an accurate assessment of the value of the water exchange rate *k*_ex_ (*k*_ex_=1/*τ*_M_).[^25c^, ^39^] The *R*_2_ values, normalized to the concentration of 1 mM (*r*_2_) and shown in Figure 3 A, increase with increasing temperature tending to reach a broad maximum that is estimated around 350 K. This indicates a changeover from a slow exchange situation, at lower temperatures, to an intermediate exchange regime at higher temperatures. The data is typically analysed using the well‐known Swift–Connick equations.[^21b^, ^39^]

A global fit of the ^1^H and ^17^O NMR data was performed using as adjustable parameters *τ*_R_, the electronic relaxation parameters *Δ* ^2^ (trace of the squared zero‐field splitting, ZFS, tensor), and ^298^ *τ*_V_ (correlation time for the modulation of the transient ZFS), the residence lifetime of the bound water molecules ^298^ *τ*_M_, the hyperfine coupling constant A_O_/*ħ*, the enthalpy of activation of *τ*_M_, Δ*H*_M_, and the activation energy of *τ*_V_, *E*_V_. The experimental data could be accurately reproduced with the parameters listed in Table 1 (a complete set of fitting parameters is reported in Table S11). The rotational correlation time (^298^ *τ*_R_) obtained for **MnL^Me^** is fully comparable with that of [Mn(EDTA)(H_2_O)]^2−^, as expected based on the similarity of their molecular sizes.^[21a]^ Furthermore, this value confirms the previous qualitative analysis of the limiting role of ^298^ *τ*_R_ on relaxivity, particularly at the magnetic fields relevant for clinical imaging. Instead, the parameters related to the relaxation of the electron spin of the Mn^2+^ ion, *Δ* ^2^ and its associated correlation time *τ*_V_, are quite different from those of [Mn(EDTA)(H_2_O)]^2−^ and [Mn(H_2_O)_6_]^2+^. This reflects the presence of somewhat different coordination environments of the metal ion in the complexes. The water exchange rate calculated for **MnL^Me^** is 5.2×10^6^ s^−1^. This value is significantly lower than that of many anionic or neutral complexes measured so far (e.g. [Mn(dpama)] and [Mn(EDTA)(H_2_O)]^2−^;[^25a^, ^25b^, ^25c^] Table S11) but it is quite similar to that reported for a structurally related complex featuring the same overall electric charge.^[16]^ The maximum value of *r*_2_ cannot be experimentally determined for this complex, but only extrapolated from the calculated curve. However, it can be used to get a rough estimate of the hydration number of **MnL^Me^**, using the method proposed by Gale et al.^[42]^ We obtained a *q* value of 1.7, confirming the presence of two inner‐sphere water molecules in the complex.

**Table 1:** Selected best‐fit parameters obtained from the analysis of the 1/*T*_1_ NMRD profiles and ^17^O NMR data of **MnL^Me^** and its BSA adduct.

Parameters	[MnL^Me^]^2+^	[Mn(dpma)]^[c]^
^298^ *r*_1_ 20 MHz [mM^−1^ s^−1^]^[a]^	5.7±0.2	5.3
^298^ *τ*_RL_ [ps]	52.0±0.1	47.8
^298^ *k*_ex_ [×10^6^ s^−1^]	5.2±0.2	3.3
Δ*H*_M_ [kJ mol^−1^]	29.7±1.2	28.1
*q*	2 ^[b]^	2

Parameters	MnL^Me^/BSA^[e]^	Mn(DPAC12A)/HSA^[d]^
^298^ *r*_1_^b^ 20 MHz [mM^−1^ s^−1^]^[a]^	21.1±0.7	15.5
*K*_A_ [M^−1^]	4.2±0.3×10^3^	1.4×10^5^
^*298*^ *τ*_RG_ [ns]^[b]^	50^[b]^	50
^*298*^ *τ*_RL_ [ps]	408±35	306
^298^ *k*_ex_ [×10^6^ s^−1^]	2.6±0.4	/
Δ*H*_M_ [kJ mol^−1^]	45.7±2.0	/
*S* ^2^	0.28±0.01	0.26
*q*	1	1^[b]^

[a] value obtained at 32 MHz, [b] fixed during the fitting procedure, [c] ref. [25b] and [d] from ref. [25a], [e] [BSA]=1.43 mM.

### Serum Protein Binding

Non‐covalent interaction of paramagnetic complexes with slowly tumbling natural biological macromolecules such as proteins enhances the *r*_1_ by lengthening the rotational correlation time *τ*_R_.[^39^, ^43^] However, the preparation of such macromolecule binding contrast agents requires multiple synthetic steps when compared to small contrast agents. Proton Relaxation Enhancement (PRE) approach has been used to investigate the non‐covalent binding interaction of **MnL^Me^** with BSA in aqueous solution at 32 MHz, neutral pH, and 298 K. The method is based on the measurement and analysis of the variation in the relaxation rate *R_1_* of the solvent water protons following the addition of increasing amounts of the protein to a 0.1 mM solution **MnL^Me^**. *R_1_* was measured before and following the addition of different amounts of BSA, covering a concentration range between ca. 0.01–1.3 mM (Figure 3 C). The observed relaxation rate, *R*_1_^obs^, increases with the progressive additions of BSA, due to the concomitant increase in the protein‐bound molar fraction of the complex and tends to an asymptotic value associated with the relaxivity of the bound complex, *r*_1_^b^ (Figure 3 C). The analysis of this isothermal binding curve in terms of PRE equations allows us to extract fairly accurate values of the relaxation of *r*_1_^b^ (21.1±0.7 mM^−1^ s^−1^) and of the term *nK*_A_ (4.2×10^3^ M^−1^), where *n* represents the number of equivalent and independent binding sites and *K*_A_ is the affinity constant.^[44]^ All the data were fitted to a 1:1 binding isotherm even though the presence of multiple low‐affinity sites on BSA cannot be strictly excluded. Association constants with very similar values, in the case of human serum albumin, have been reported for macrocyclic Mn^II^ complexes functionalized with benzyl groups.^[45]^ The NMRD profiles of the BSA adduct was measured at 298 K (Figure 3 B) for a diluted solution (0.1 mM) of **MnL^Me^** in the presence of 1.3 mM BSA. These conditions ensure more than 99 % binding of **MnL^Me^** to the protein.

In several cases, the interaction of bishydrated complexes (*q*=2) of Gd^III^ with the protein was accompanied by a reduction in the state of hydration and the exchange rate of the inner sphere water molecule(s).^[46]^ To better evaluate these aspects and obtain an accurate estimate of *k*_ex_, we measured the temperature dependence of ^17^O NMR *R*_2_ in a solution of **MnL^Me^** (0.12 mM) containing an excess of BSA (1.43 mM) in HEPES. The temperature dependency of the *r*_2_ values is reported in Figure 3 A and visually clearly indicates the lower hydration state of the complex bound to the protein, as testified by a marked reduction of *r*_2_ values at all explored temperatures respect to what observed for the free chelate. The ^1^H and ^17^O NMR data were analysed as in the previous case. The NMRD profile was fitted only in the high‐field region (>3 MHz) because of the known limitations of the SBM theory for slowly tumbling systems at low magnetic field strengths.^[47]^ The NMRD profile analysis was performed using the Lipari‐Szabo approach for the description of rotational dynamics.^[48]^ This model takes into consideration the occurrence of the anisotropic motion condition, which is attributable to a certain degree of local rotation at the binding site superimposed on the global rotational motion of the protein. The two types of motions are characterized by different correlation times: *τ*_RL_ and *τ*_RG_, respectively. The degree of correlation between the two types of motion is expressed by the order parameter *S* ^2^, which takes values between 0 (completely independent motions) and 1 (completely correlated movements). The data is reproduced satisfactorily using the value of 1 (Figures 3 A,B, and Table 1) for *q*. The reduction of *q* from 2 to 1 implies the formation of a ternary complex with the protein, with a displacement of a water molecule from a coordinating group of BSA, easily an acetate.

Derivatives of [Gd(DO_3_A)(H_2_O)] with pendant hydrophobic chain have been reported to form ternary adducts with HSA characterized by completely analogous *K*_A_ values and water displacement from the protein. Even in this case, we have confirmed the value of *q* from the maximum value of ^17^O NMR *r*_2_ calculated from the best‐fit curve (*q*≈0.9). The data analysis also indicates a 2‐fold slower *k*_ex_ compared to the free complex (*k*_ex_=2.6×10^6^ s^−1^; *τ*_M_=378 ns).

This decrease in the water exchange rate may be due to a lower accessibility of bulk water to the metal ion at the binding site, or to the involvement of water bound in hydrogen bonds with proximate groups of the protein.

The analyses of the NMRD profile using the Lipari‐Szabo model (Table 1) indicate that a high degree of local flexibility is responsible for the relatively low relaxivity of the adduct formed between **MnL^Me^** and BSA, consistent with the low values of *τ*_RL_ and *S* ^2^. To further validate the protein binding affinity of **MnL^Me^** and the number of binding sites available in BSA for **MnL^Me^**, fluorescence spectral titrations of BSA versus **MnL^Me^** and Mn^2+^ (Figures 3 D and S18) in 50 mM HEPES buffer at pH 7.4, 298 K were carried out. As expected, the initial fluorescence intensity of BSA (50 μM) at 355 nm was dramatically decreased upon the addition of an equivalent of **MnL^Me^** (0–150 μM) and became relatively constant to significant excess. The linear relationship sharply deviated when plotting *F*_0_/*F* vs. [**MnL^Me^**], and an upward curvature was observed. This is due to the interaction of **MnL^Me^** with BSA in its ground state (static quenching) and its first excited singlet state (dynamic quenching) as well. The average association constant *K_A_* and the number of binding sites (n) in BSA (from modified Scatchard plot analysis)^[49]^ are found to be 4.02×10^3^ M^−1^ and 1.0, respectively, correlating with the PRE experiment. In contrast, there is no considerable fluorescence quenching upon addition of Mn^2+^ (Figure S18), thus suggesting that the ligand moiety in **MnL^Me^** is essential for the BSA binding.

In support of the previous experiments, we also performed *T*_1_‐ and *T*_2_‐ weighted phantom imaging at 7.0 T with **MnL^Me^** solutions at different concentrations, with and without BSA (Figures 3 E and S19). Results show that the *T*_1_‐contrast enhancement was only modest in the samples containing **MnL^Me^** in solution but highly enhanced in the presence of 0.6 mM BSA (Figure 3 E). Moreover, as expected, we observed a superior *T*_2_‐contrast modulation from **MnL^Me^** at 7 T (Figure S19). This is due to the favorable transverse paramagnetic relaxation properties of Mn^2+^ at a high magnetic field compared to Gd^3+^.^[42]^

### In Vivo MRI Studies

**Figure 4:**
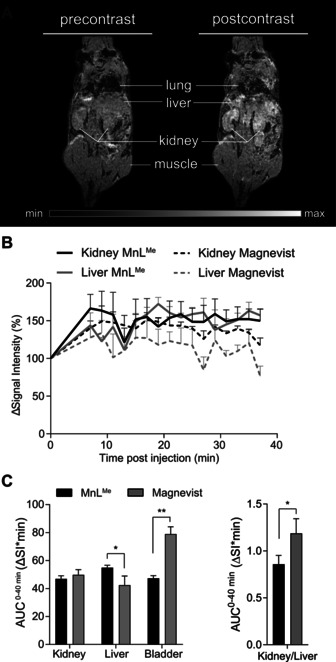
A) Representative *T*_1_‐weighted MR images illustrate contrast enhancement in the kidneys, liver, heart, and bladder after application of **MnL^Me^** at a clinical dose (right panel) in comparison to pre‐contrast imaging (left panel). B) Percentage of signal intensity enhancement normalized to muscle for all organs of interest revealed a comparable dynamic excretion profile for **MnL^Me^** and Magnevist in liver and kidneys (mean ± SEM, *n*=3 for **MnL^Me^**, *n*=3 for Magnevist^®^). C) (left panel) Total signal intensity over time of **MnL^Me^** represented as the area under the curve (AUC^0–40^ min (Δ*S*I*min)) is significantly higher in the liver and lower in the bladder compared to Magnevist (mean ± SEM, *n*=3 for **MnL^Me^**, *n*=3 for Magnevist). (right panel) The kidney‐to‐liver ratio of total signal intensity represented as area‐under‐the‐curves (AUC^0–40^ min (Δ*S*I*min)) is significantly lower for **MnL^Me^** compared to Magnevist^®^ (mean values ± variance, *n*=3 for **MnL^Me^**, *n*=3 for Magnevist, statistics: Student's t‐test, *p‐value <0.05, **p‐value <0.01).

Dynamic *T*_1_‐weighted MRI measurements were performed in healthy mice to evaluate the potential of **MnL^Me^** as an MRI CA. Figure 4 A shows the pre‐ and post‐contrast (7 min) coronal MR images following the intravenous injection of 0.1 mmol kg^−1^ **MnL^Me^**. Elimination is observed through the liver, kidneys, and the bladder. Contrary to previously reported MnCAs, no significant contrast enhancement was found in the gallbladder.^[50]^ Regardless, the distribution of **MnL^Me^** followed similar pathways previously observed for small Mn‐ and Gd‐based complexes.[^28b^, ^29a^, ^50^] For comparison, we have performed dynamic MRI studies with the commercially available Gd‐based agent [Gd(DTPA)(H_2_O)]^2−^ (Magnevist^®^) (bolus of 0.1 mmol kg^−1^).[^17a^, ^51^] Figures 4 B and S20 clearly show that **MnL^Me^** provides similar normalized change in signal intensity (Δ*S*I) values in the kidneys over the 40 min imaging time, denoting the fast clearance of the agent.

The higher contrast produced by **MnL^Me^** in the liver over the 40 min suggests a slower clearance through the liver. This is also confirmed by lower contrast in the bladder due to the gradual elimination. In Figure 4 C, we compared the area‐under‐the‐curve (AUC) for each tissue over the imaging time of 40 minutes post‐injection (AUC^0–40 min^). Results showed that both compounds indeed present similar excretion pathways; however, the kidney‐to‐liver AUC^0–40 min^ ratio for both agents indicates a slower clearance of **MnL^Me^** (Figure 4 C). Although not reaching statistical difference, data also suggest a longer half‐life in the blood, as observed by the higher Δ*S*I values on the left ventricle of the heart (Figure S20). This feature enables for a longer image acquisition window after bolus injection and avoids the use of highly concentrated doses of CAs by constant infusion.^[52]^ Since albumin is the most abundant protein in the blood with an average concentration of 0.6 mM, any enhanced contrast observed in the *T*_1_‐weighted imaging results from specific **MnL^Me^** binding to murine serum albumin in vivo. While further studies are necessary to test the fraction of **MnL^Me^** bound to serum albumin in vivo, the presented in vivo data indicates safe use of **MnL^Me^** as a specific MRI blood pool contrast agent.

## Conclusion

We have designed and synthesized a new pentadentate Schiff‐base type Mn^2+^ complex (**MnL^Me^**) in a one‐pot reaction, interrogated its structural, stability, kinetic inertness, relaxometric properties and explored its biodistribution in healthy mice using in vivo MRI. This is the first example to date of template to MRI contrast agent synthesis. This non‐macrocyclic Mn^2+^ complex displays relatively enhanced water proton relaxivity (*r_1_*) 4.88 and 5.2 mM^−1^ s^−1^ at 400 MHz (9.4 T) and 64 MHz (1.5 T), respectively, compared to the reported acyclic mono and bishydrated Mn^2+^ and Gd^3+^ complexes. The pH‐dependent relaxivity and zinc transmetallation kinetics study results confirm that the complex is stable at neutral pH conditions. The **MnL^Me^** shows a moderate binding affinity with BSA, which results in enhanced relaxivity upon binding due to the decreased molecular rotation.

The concentration‐dependent signal enhancement in the phantom *T*_1_‐weighted MR images suggests that the capability of **MnL^Me^** is comparable to the clinically available acyclic Gd^3+^ complex [Gd(DTPA)(H_2_O)]^2−^ (Magnevist^®^). We also have demonstrated this in vivo in healthy mice by MRI. The clearance of **MnL^Me^** follows a renal/hepatic elimination pathway. **MnL^Me^** is excreted intact into the bladder and shows a delayed clearance from the blood as observed by the continuous high Δ*S*I on the left ventricle of the heart. As in vitro data indicates that **MnL^Me^** interacts with albumin and first in vivo experiments yielded high Δ*S*I comparable to [Gd(DTPA)(H_2_O)]^2−^ (Magnevist^®^), **MnL^Me^** will be further investigated as a potential MRI blood pool contrast agent.

## Conflict of interest

The authors declare no conflict of interest.

## Supporting information

As a service to our authors and readers, this journal provides supporting information supplied by the authors. Such materials are peer reviewed and may be re‐organized for online delivery, but are not copy‐edited or typeset. Technical support issues arising from supporting information (other than missing files) should be addressed to the authors.

Supplementary
