# Supplementary material for: A Single‐Pot Template Reaction Towards a Manganese‐Based T 1 Contrast Agent
Source: Angew Chem Weinheim Bergstr Ger. 2021 Apr 1;133(19):10831–9. doi: 10.1002/ange.202100885 (PMC10947048; doi:10.1002/ange.202100885)
Supplement: Supplementary file 1 — Supplementary [file ANGE-133-10831-s001.pdf]

Angewandte Chemie
Eine Zeitschrift der Gesellschaft Deutscher Chemiker

Supporting Information

A Single-Pot Template Reaction Towards a Manganese-Based T_1 Contrast Agent

Sellamuthu Anbu[+], Sabrina H. L. Hoffmann[+], Fabio Carniato, Lawrence Kenning, Thomas W. Price, Timothy J. Prior, Mauro Botta, Andre F. Martins,* and Graeme J. Stasiuk**

ange_202100885_sm_miscellaneous_information.pdf

Supporting Information
©Wiley-VCH 2019
69451 Weinheim, Germany

Abstract: Manganese-based contrast agents (MnCAs) emerged as suitable alternatives to gadolinium-based contrast agents (GdCAs). However, due to their kinetic lability and laborious synthetic procedures, only a few MnCAs have found clinical MRI application. In this work, we have employed a highly innovative single pot template synthetic strategy to develop a MnCA, **MnL^{Me}**, and studied the most important physicochemical properties *in vitro*. **MnL^{Me}** displays optimized, $r_1$relaxivities at both medium (20 and 64 MHz) and high magnetic fields (300 and 400MHz) and an enhanced r_1^b = 21.1 mM^{-1}s^{-1} (20 MHz, 298 K, pH 7.4) upon binding to BSA (K_a = 4.2 × 10^3 M^{-1}). *In vivo* studies show that **MnL^{Me}** is cleared intact into the bladder through hepatobiliary excretion and a prolonged blood half-life compared to the commercial GdCA Magnevist. **MnL^{Me}** shows great promise as a novel MRI contrast agent.

DOI: 10.1002/anie.2016XXXXX

Table of Contents

Experimental Procedures

Figures. S1-S2. ^1H and ^{13}C-NMR spectral profiles of **ZnL^{Me}**

Figure S3. Absorption spectra of **MnL^{Me}** and **ZnL^{Me}**

Figure S4-S5. ESI–Mass spectral profiles of **MnL^{Me}** and **ZnL^{Me}**

Figure S6. X-Ray crystal structure of **ZnL^{Me}.** Bound water (O3) forms two hydrogen bonds to unbound water and chloride. The N−H functions of the ligand form hydrogen bonds to unbound water and chloride. The bound chloride acts as a hydrogen bond acceptor to unbound water, and O1 also acts as a hydrogen bond acceptor to unbound water. Both complexes are arranged in layers parallel to (101). Within and between the layers, these hydrogen bonds assemble the complexes and unbound species into a 3−D hydrogen-bonded network.

Figure S7. Crystal packing diagram of **MnL^{Me}**

Figure S8. The plot of $1/T_1$ vs. [**MnL^{Me}**] and T_1-weighted phantom images of **MnL^{Me}**

Figure S9. Zn^{2+}-transmetallation of **MnL^{Me}** monitored by relaxation rate; R_1 ($1/T_1$, s^{-1}) vs. Time

Figure S10. Time-dependent relaxivity of **MnL^{Me}**

Figure S11. Time-dependent relaxivity and Zn^{2+} (25 mM) transmetallation of1 mM solutions of **MnL^{Me}**, $[Mn(EDTA)]^{2-}$, $[Mn(DTPA)]^{3-}$ and $[Gd(DTPA)]^{2-}$ in 50 mM MES buffer at pH 6.0, 400 MHz (9.4 T), 298 K.

Figure S12. Chelating stability of **MnL^{Me}** was monitored by change in the absorption spectral intensity as a function of time. Absorption spectra of **MnL^{Me}** (50μM) obtained over 10 days (A), Change in the absorption intensities at 213 and 275 nm (B), and the ratio of Abs_{275}/Abs_{213} as the function of time (C).

Figure S13. Change inrelaxivity of **MnL^{Me}** (1 mM) in 50 mM aqueous (phosphate/phosphoric acid (pH 3.0), MES (pH 5.2 and 6.0), HEPES (pH 7.3), Potassium phosphate/KOH (pH 8.5), NH_4Cl/NH_3 (pH 9.3 and 10.0) buffer solutions, 400 MHz (9.4 T), 25 °C.

Figure S14. Transchelation of **MnL^{Me}**. (a) no transchelation observed in competition with 25 molar equivalents of DTPA (b), nor with 25 molar equivalents EDTA (c). Solutions were incubated for 1 hour with the competing ligands.

Figure S15. Figure S15. LC-MS of mouse urine samples. LC-MS analysis of pure **MnL^{Me}**(a) and a urine sample from a healthy mouse after intravenous administration of a clinical dose of **MnL^{Me}** (b). A control urine sample without prior **MnL^{Me}** administration (c) and a urine sample spiked with **MnL^{Me}** (d) on an Agilent LC-MS system (LC: 1200 series, MS: 6120 quadrupole). MS chromatogram (e) of the pure **MnL^{Me}** extracted at *m/z*+ 329.1 and the ESI-MS spectra at 520 sec (8.6 min). At 40 min post intravenous **MnL^{Me}** (0.1 mmol/kg bodyweight, 2.5×10^{-6} M) injection, 24 % of the contrast agent was excreted intact into the bladder. The asterisk (*) marks a signal from the eluent system.

Figure S16. ^1H NMRD profiles of **MnL^{Me}** at different temperatures

Figure S17. Temperature dependence of the relaxivity for **MnL^{Me}**

FigureS18. Fluorescence spectral profile of 50 μM BSA protein in 50 mM HEPES buffer (pH 7.4, 298 K) with increasing quantity of aq. **Mn^{2+}** solution (0–250 μM, λ_{ex} = 280 nm).

Figure S19. T_1 and T_2-weighted phantom imaging of **MnL^{Me}** and Magnevist. (A) T_1-weighted imaging of 0.1-0.5 mM MnLMe without and with 0.6 mM BSA in 50 mM HEPES (first and second row, respectively) illustrates highly enhanced contrast enhancement in **MnL^{Me}** samples with BSA in comparison to **MnL^{Me}** samples without BSA. Compared to 0.1-0.5 mM Magnevist without and with 0.6 mM BSA in 50 mM HEPES (third and fourth row, respectively), overall contrast enhancement of **MnL^{Me}** samples is lower. Images were recorded at 7 T and 298 K with a flip angle of 10°. (B) T_2-weighted imaging of 0.1-0.5 mM **MnL^{Me}** without and with 0.6 mM BSA in 50 mM HEPES (first and second row, respectively) shows superior T_2 contrast modulation of **MnL^{Me}** in comparison to 0.1-0.5 mM Magnevist without and with 0.6 mM BSA in 50 mM HEPES (third and fourth row, respectively).

Figure S20. Biodistribution of Magnevist and **MnL^{Me}**: (A) Representative images of Magnevist®-induced contrast enhancement *in vivo*. (B) Total signal intensity over time of **MnL^{Me}** represented as the area under the curve ($AUC^{0\text{-}40\ min}$ (ΔSI*min)) was comparable to Magnevist in all analyzed organs (mean ± SEM, n = 3 for **MnL^{Me}**, n = 3 for Magnevist®). Percentage of signal intensity enhancement normalized to pre-injection over time for all organs of interest is given in (C) for **MnL^{Me}** and (D) for Magnevist®.

Scheme S1. MnL^{Me} binds with bovine serum albumin (BSA), resulting in an enhanced relaxivity due to the decreased molecular rotation.

Tables S1-S10. Crystallographic data for **MnL^{Me}** and **ZnL^{Me}**

Tables S11 and S12. Parameters from the analysis of ^1H NMRD data.

Experimental Procedures

Materials and Measurements. All of the materials and reagents for the synthesis of mononuclear Mn^{2+} and Zn^{2+} complexes (**MnL^{Me}** and **ZnL^{Me}**) were purchased from Sig-ma-Aldrich (Dorset, UK) and used without further purification. Solvents were purchased from VWR (Leicestershire, UK). IR spectra were recorded on a Perkin Elmer Rx FTIR x2 with diamond ATR, DRIFT attachment. ^{1}H-NMR and T_1 measurements were carried out on a JEOL JNM-LA400 Spectrometer (400 MHz). Electronic absorption spectral titration experiments were done using a ThermoFisher Scientific Evolution 300 UV-Vis spectrophotometer, and the fluorescence emission studies were carried out on a Horiba Fluoromax-4P spectrofluorometer. High resolution mass spectrometry (HRMS) spectra of the compounds were recorded on Advion MS SOP electrospray ionization (ESI) spectrometer. The pH measurements were carried out by using a Jenway 3520 digital pH meter with a Mettler-Toledo 51343160 glass electrode.

Synthesis of Mn^{2+} complex MnL^{Me} (1). 2,6-diacetylpyridine (0.163 g, 1.0 mmol) was added to 60 mL of methanol (MeOH), and solid $MnCl_2$ (0.121 g, 0.96 mmol) was added and stirred for an hour at 60 °C. To this mixture, a methanolic solution (20 mL) of acetohydrazide (0.148 g, 2.0 mmol) was added dropwise. The mixture turned slowly from colorless to pale yellow and was refluxed overnight, and hot filtered. The solvent was evaporated in air, and the resultant bright yellow solid was washed with little amounts of isopropanol/diethyl ether mixture and dried. The obtained water-soluble yellow solid was recrystallized from water and MeOH (2:1, v/v) mixture. Isolated yield: bright yellow block crystals, 0.32g (91%). Anal. Calcd for $[Mn(L^{Me})Cl(H_2O)]$•$Cl.2H_2O$: C, 34.30; H, 5.09; N, 15.39. Found: C, 34.31, H, 5.07, N, 15.35. FTIR (neat, cm^{-1}): 3428b (N−H), 1637s (C=N), 1609s (C=O), 1026s (−N−N). MS (ESI$^+$) in CH_3OH: m/z 365.04 ($[Mn(L^{Me})(Cl^-)]^+$), 329.07 07 ($[Mn(L^{Me}\text{-}H^+)]^+$). . HRMS: calcd. for $C_{13}H_{17}Cl_1N_5O_2Mn_1$ 365.0446 found 365.0451.

Synthesis of Zn^{2+} complex ZnL^{Me} (2). The Zn^{2+} complex was synthesized by following the above procedure with 24 h refluxing using $ZnCl_2$ (0.126 g, 0.96 mmol) instead of $MnCl_2$. Isolated yield: Pale yellow crystals, 0.18 g (49%). Anal. Calcd for $[Zn(LMe)Cl(H_2O)]$•$Cl.2H_2O$: C, 33.53; H, 4.90; N, 15.04. Found: C, 33.30, H, 4.91, N, 14.84. ^{1}H NMR (D_2O, 400 MHz) δ (ppm): 8.2 (br, 1H, H-Pyridine), 8.00 (d, 2H, J = 10.0 Hz, H-Pyridine), 2.44 (br, 6H, H-methyl) and 2.23 (br, 2H, 6H-methyl). ^{13}C NMR (D_2O, 100 MHz) δ (ppm): 12.60, 19.69, 124.50, 143.13, 147.60, 148.09 and 173.33. FTIR (neat, cm^{-1}): 3453s (N−H), 1642s (C=N), 1614s (C=O), 1037s (−N−N). MS (ESI$^+$) in CH_3OH: m/z 374.18 ($[Zn(L^{Me})(Cl^-)]^+$), 339.14 ($[Zn(L^{Me}\text{-}H^+)]^+$). ES-MS: calcd. for $C_{13}H_{17}Cl_1N_5O_2Zn_1$ 374.04 found 374.18.

X-Ray Data Collection and Reduction. Single crystal X ray diffraction data were collected in series of ω-scans using a Stoe IPSD2 image plate diffractometer utilizing monochromated Mo radiation (λ = 0.71073 Å). Standard procedures were employed for the integration and processing of the data using X-RED.[1] Samples were coated in a thin film of perfluoropolyether oil and mounted at the tip of a glass fiber located on a goniometer. Data were collected from crystals held at 150 K in an Oxford Cryosystems nitrogen gas cryostream.

Crystal structures were solved using dual space methods implemented within SHELXT.[2] Completion of structures was achieved by performing least squares refinement against all unique F2 values using SHELXL-2018.[3] All non H atoms were refined with anisotropic displacement parameters. Hydrogen atoms were placed using a riding model. Where the location of hydrogen atoms was obvious from difference Fourier maps, C-H and O-H bond lengths were refined subject to chemically sensible restraints.

Pseudosymmetry in the crystal structures of MnL^{Me} and ZnL^{Me}. Both structures crystallise in $P2_1/c$ but are close to being body centred. Each structure solves and refines very satisfactorily in this space group and the refinement is stable. In neither case is there convincing evidence for a body-centring translation although the symmetry of the primitive cell generates a pseudo-body-centred array of the complexes. However, the strict bodycentring translation is not obeyed; complexes related by the pseudo translation are rotated by approximately 180 degrees about the Zn1-N1 bond.

The diffraction data below demonstrate that the true symmetry is P not I.

Observed diffraction data for MnL^{Me}:

Reflections with h+k+l=2*n*:

5688 refs, mean (I/σ_I) = 10.4 and of these 2483 have I> $4\sigma_I$

Reflections with h+k+l=2*n*+1: 5426 refs, mean (I/σ_I) = 4.9 and of these 1676 have I> $4\sigma_I$.

The data consistent with bodycentring are thus weak but not systematically absent.

Observed diffraction data for ZnL^{Me}

Reflections with h + k + l = 2*n*: 6441 refs, mean (I/σ_I) = 7.5 and of these 2488 have I > $4\sigma_I$

Reflections with h + k + l = 2*n*+1: 6441 refs, mean (I/σ_I) = 1.7 and of these 741 have I > 4 σ_I.

The data consistent with bodycentring are thus weak but not systematically absent.

Relaxometry studies. The longitudinal relaxation times (T_1) and water proton relaxation rates ($r_1 = 1/T_1$) of the **MnL^{Me}** were measured on a JEOL JNM-LA 400 Spectrometer (400 MHz). The T_1 values were obtained by the inversion-recovery method (180º − τ − 90º). The relaxivity of the complex was determined by different concentrations of **MnL^{Me}** (1.0 to 5.0 mM) at pH = 7.3 (50 mM HEPES buffer, 0.15 M NaCl, 298 ± 0.2 K). The water proton relaxivity r_1 of the **MnL^{Me}** was determined from the slope of the plot of $1/T_1$ vs. [**MnL^{Me}**]. Stock solutions of **MnL^{Me}** (10 mM) and BSA were prepared in MilliQ water. The concentration of BSA was determined spectroscopically based on the tryptophan absorbance at 280 nm (ε_{280} = 43824 M^{-1} cm^{-1}) in water. The hydration state of the metal was empirically calculated by applying the following equations (1) and (2):[4]

$$y = 9.16\left\{1 - exp\left(-2.97 \times FW \times 10^{-3}\right)\right\} \quad \ldots.(1) \qquad q_{calc} = \frac{r_1}{y} \quad \ldots.(2)$$

where, FW = 366.28 g/mol; y = 6.05; r_1 = 13.9 mM^{-1} s^{-1} and q = 2.3.

^1H Nuclear Magnetic Relaxation Dispersion (NMRD) profiles were measured on a Fast-Field Cycling (FFC) Stelar Smart Tracer Relaxometer over a continuum of magnetic field strengths from 0.00024 to 0.25 T (corresponding to 0.01-10 MHz proton Larmor Frequencies). Additional data points in the range 20-120 MHz were obtained with a High Field Relaxometer (Stelar) equipped with the HTS-110 3T Metrology Cryogen-free Superconducting Magnet. The measurements were performed using the standard inversion recovery sequence (20 experiments, 2 scans) with a typical 90° pulse width of 3.5 μs and the reproducibility of the data was within ± 0.5%. The temperature was con-trolled with a Stelar VTC-91 heater airflow equipped with a copper-constantan thermocouple (uncertainty of ± 0.1 K). The Mn(II) concentration was estimated by ^1H-NMR (Bruker Advance III Spectrometer equipped with a wide bore 11.7 T magnet) measurements using Evans's method.[5] The hydration number q was adjusted to 2; the distance between the metal ion and the protons of the bound, r_{MnH}, and *outer sphere*, a_{MnH}, water molecule were set to 2.74 and 3.6 Å, respectively. The value of the distance r_{MnH} is consistent with the average distances obtained (r_{MnH} = 2.7373 Å) from X-ray crystal structure data of **MnL^{Me}**.

^{17}O NMR

MnL^{Me}: ^{17}O NMR spectra were acquired on a Bruker Avance III spectrometer (11.7 T) using a 5 mm probe under temperature control. An aqueous solution of the Mn(II) chelate (2.3 mM) in HEPES buffer (pH = 7.4) containing 2.0% ^{17}O-enriched water (Cambridge Isotope) was prepared and analysed. The transverse relaxation rates were measured from the signal width at half-height, as a function of temperature in the 278-350 K range. A diamagnetic blank solution containing acidified water (pH = 3.0) enriched with $H_2{}^{17}O$ (2.0%) was used as reference. Notably, the observed transverse relaxation rates R_2 depend on the rate of water exchange at 298 K, $^{298}k_{ex}$, and its activation enthalpy ΔH_M, the electronic relaxation time $^{298}T_{2e}$, and the ^{17}O hyperfine coupling constant $A_O/\hbar$.

MnL^{Me}/BSA: the same procedure was applied to the BSA-bound complex. A 0.12 mM solution of the complex and 1.43 mM of BSA in HEPES containing 2.0% ^{17}O-enriched water (Cambridge Isotope) was prepared. The diamagnetic blank solution was prepared by dissolving BSA (1.43 mM) in HEPES containing 2.0% of the ^{17}O isotope (Cambridge Isotope).

The hydration state of Mn(II) (q) was calculated by using the empirical equation[6] reported below:

$$q = r^{o}_{2max}[H_2O]\left(\frac{2}{\sqrt{\frac{S(S+1)}{3}\frac{A_o}{\hbar}}}\right) \cong \frac{r^{o}_{2max}}{510}$$

where, $r_2°_{max}$ is the maximum ^{17}O transverse relaxivity and $A_O/\hbar$ is the hyperfine coupling constant.

MR Imaging. Phantoms of 0.1-0.5 mM **MnL^{Me}** in 50 mM HEPES buffer (pH 7.4) and 0.1-0.5 mM **MnL^{Me}** with 0.6 mM BSA in 50 mM HEPES buffer (pH 7.4) were prepared in a customized 5 × 5 well-plate (0.25 mL capacity). Phantom and in vivo MR imaging were acquired on a 7 T preclinical MR scanner (Bruker BioSpec 70/30, Bruker BioSpin, Ettlingen, Germany) using an 86-mm diameter ^1H transceiver volume coil (Bruker). The sample temperature was regulated with a heated pad system (Medres medical research GmbH, Cologne, Germany) and monitored with corresponding temperature probe and by ^1H spectroscopy (PRESS) on ethylene glycol. T_1-weighted MR images of **MnL^{Me}** were acquired with a standard 3D T1-FLASH sequences and the following parameters: TE = 2.64 ms, TR = 8.78 ms, flip angle 30°, FOV 60×40×21.21 mm^3, image size 272×136×96, slice thickness 21.12 mm, resolution 0.220×0.294×0.220 mm^3, 1 average with a total acquisition time of 1 min 54 s. For comparison of **MnL^{Me}** to Magnevist in T_1-weighted imaging, the flip angle was reduced to 10°. T_1 maps were acquired with a 2D RAREVTR sequence (TE 6.705 ms, FOV 50×50 mm^2, image size 128×128, resolution 0.390×0.390 mm^2, total scan time: 12 min 52 s) with 15 TR values of 50, 100, 200, 300, 400, 500, 600, 700, 800, 900, 1000, 1500, 2000, 2500 and 3000 ms. T_2-weighted images were acquired with a standard 2D spin-echo sequence and the following parameters: TE 8.993 ms, TR 4253.613 ms, FOV 60×40 mm^2, image size 200×134, resolution

0.300×0.298 mm², 1 average with a total acquisition time of 1 min 8 s. T_2-maps were acquired with a standard MSME sequence, variable TEs of 8-200ms in 8 ms steps and the following parameters: TR 2000 ms, FOV 50×50 mm², image size 128×128, resolution 0.300×0.298 mm², total scan time 4 min 16 s.

For in vivo imaging, healthy female C56BL/6 mice were anesthetized with 1.5 % isoflurane in pure oxygen. For the application of the MR contrast agents, a catheter was placed in a lateral tail vein. T_1-weighted pre- and post-contrast in vivo MR images were acquired with a 3D FLASH-sequence and the following parameters: TE = 2.64 ms, TR = 8.78 ms, flip angle 10°, FOV 75×35×21.21 mm³, image size 272×136×96, slice thickness 21.12 mm, resolution 0.276×0.257×0.220 mm³, total acquisition time 1 min 54 s. After the pre-contrast acquisition, **MnL^{Me}** (n = 3) or Magnevist (n = 3) were applied at a final dose of 0.1 mmol/Kg body weight and allowed to distribute for 5 min before 16 post-contrast images were acquired back-to-back. For data analysis in PMOD (PMOD Technologies LCC, Bruker), fixed volumes of interest were placed in the kidneys, liver, heart, bladder, and thigh muscle. Post-contrast values were normalized to pre-contrast, and the resulting percent signal chance represented over time. The area under the curve was measured over a period of 1 – 40 minutes post-contrast administration and plotted using GraphPad Prism 7. Statistical significance was evaluated using unpaired two-tailed t-tests, p-values < 0.05 were considered significant. All animal procedures were con-ducted following German federal regulations on the use and care of experimental animals and approved by the local authorities (Regierungspräsidium Tübingen).

HPLC strategies. Liquid chromatography-mass spectrometry (LC-MS) was performed using an Agilent LC-MSD system (LC: 1200 series, MS: 6120 quadrupole) and Daly conversion dynode detector with UV detection at 220, 254 and 280 nm and used the following method to analyse the samples. Luna C18 column (250 × 2 mm), eluent A: CH_3CN, eluent B: NH_4OAc buffer (pH 7.0, 298 K), gradient (0–95% CH_3CN over 18 min, flow rate 0.3 mL/min. Urine samples were preprocessed with a 10 kDa molecular weight cut off filter before injection onto the column.

NMR titrations. NMR spectra for transmetalation of **MnL^{Me}** and $[Gd(DTPA)(H_2O)]^{2-}$ (Magnevist®) with 1 and 25 molar equivalents Zn^{2+} were acquired on a 600 MHz Bruker Avance III spectrometer at 298 K. T_1 relaxation was measured via an inversion recovery experimentusing 8 inversion times (0.05, 0.075, 0.1, 0.25, 0.35, 0.5, 0.75 and 2 s) before and after addition of Zn^{2+}. T_2 relaxation was measured using a Carl-Purcell-Meiboom-Gill spin-echo experiment. Relaxivity values (r_1, r_2) were calculated for each transmetalation experiment from the previously determined concentration. The concentration of paramagnetic complexes in solution was determined from bulk magnetic susceptibility shift in NMR spectra.[5]

Results and Discussion

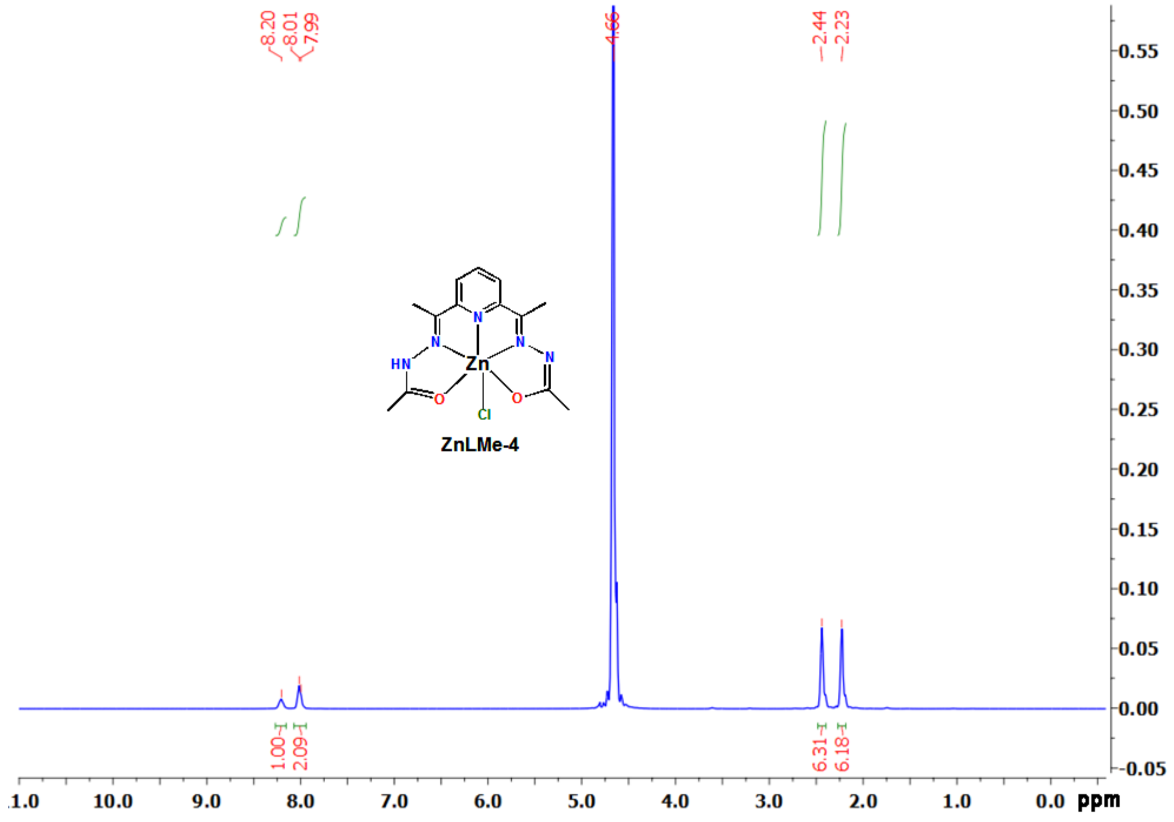

Figure S1.^{1}H-NMR spectrum of Zn^{2+} complex **ZnL^{Me}** recorded in D_2O.

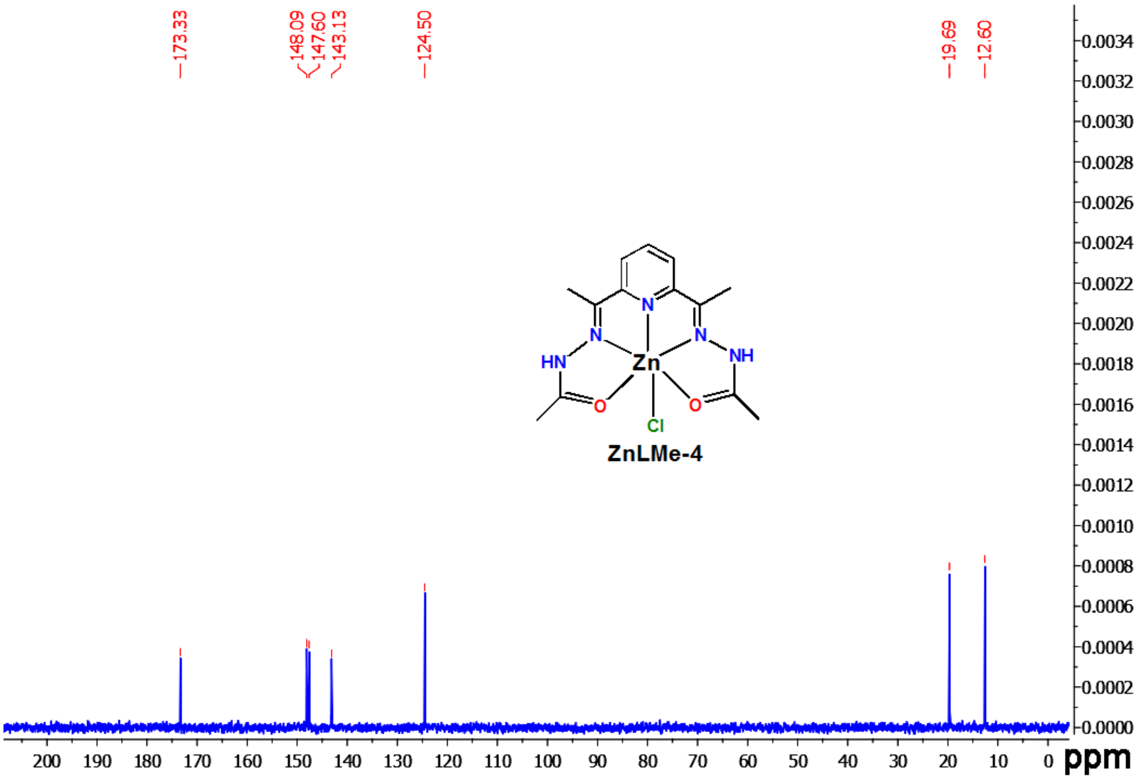

Figure S2.^{13}C-NMR spectrum of Zn^{2+} complex **ZnL^{Me}** recorded in D_2O.

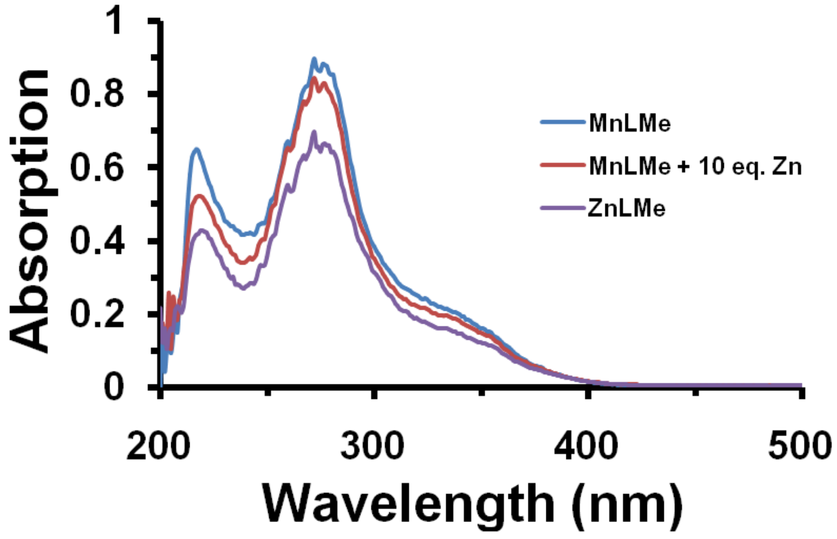

Figure S3. Absorption spectra of **MnL^{Me}**, **MnL^{Me}** + 10 eq. Zn^{2+} and **ZnL^{Me}** (50 mM) recorded in 50 mM HEPES buffer at pH = 7.3.

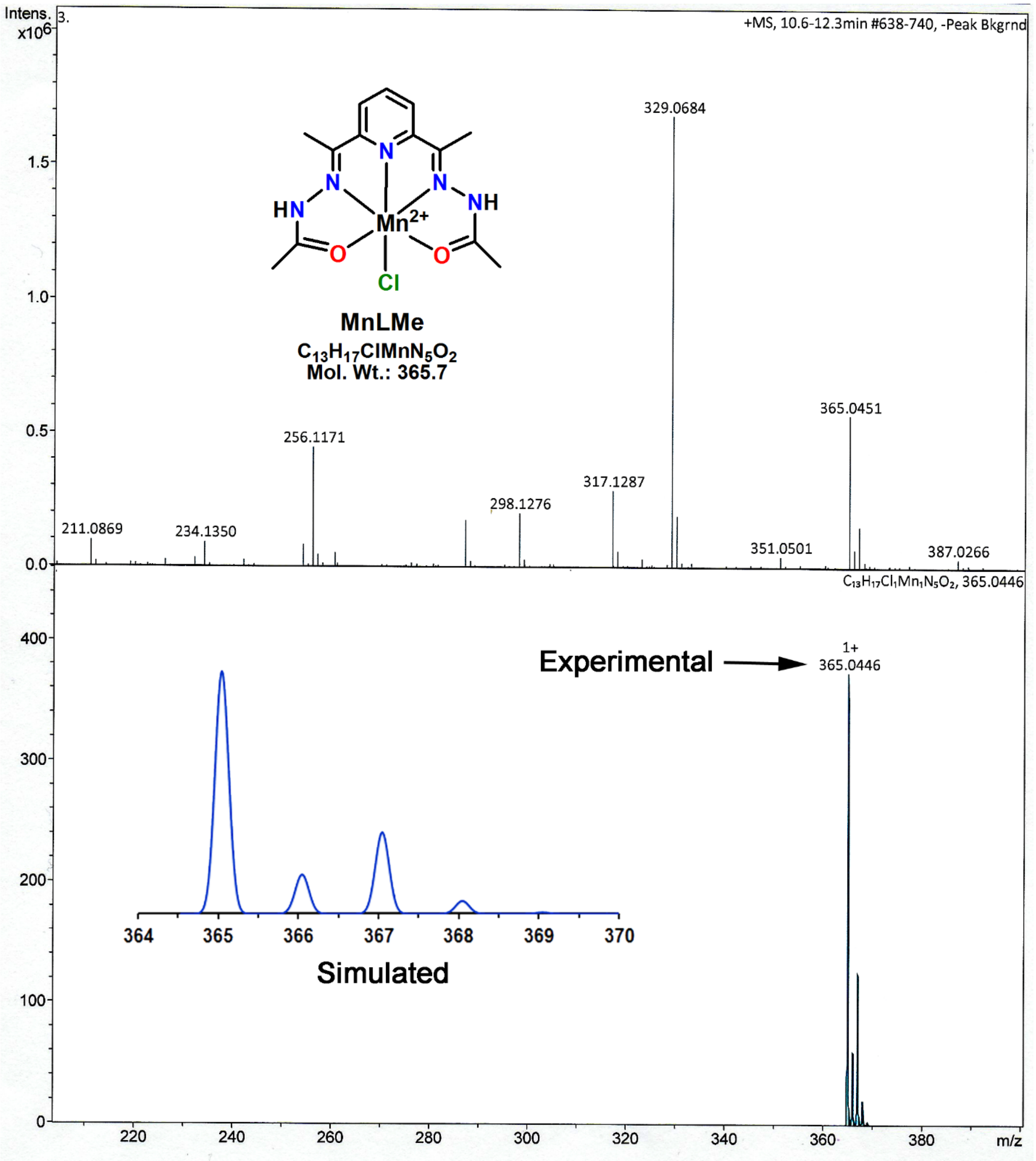

Figure S4. HRMS (ESI$^+$) spectrum of **MnL^{Me}** recorded in CH_3OH.

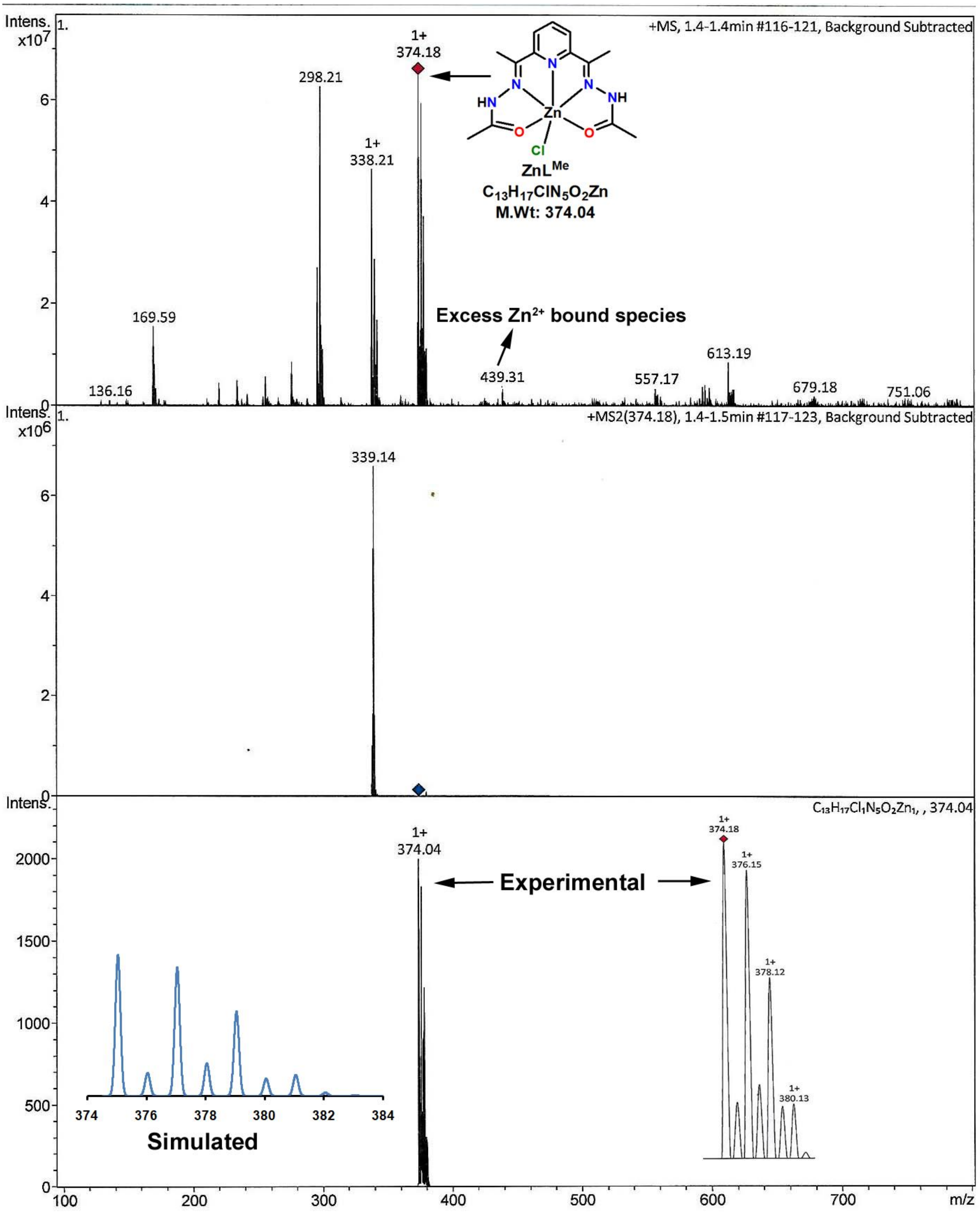

Figure S5. ESI+ spectrum of **ZnL^{Me}** recorded in CH_3OH.

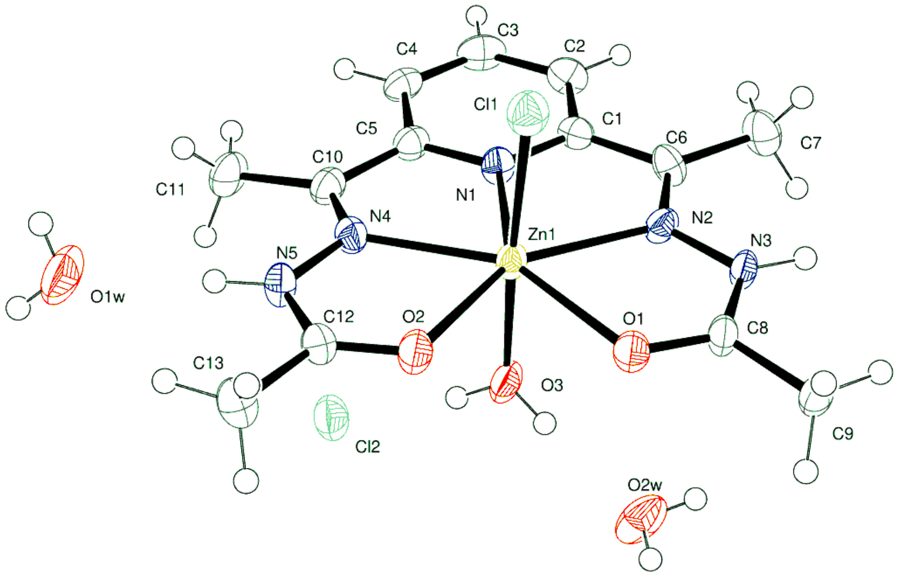

Figure S6. **ORTEP diagram of** ZnL^{Me} **drawn at 50% ellipsoid level.** Bound water (O3) forms two hydrogen bonds to unbound water and chloride. The N−H functions of the ligand form hydrogen bonds to unbound water and chloride. The bound chloride acts as a hydrogen bond acceptor to unbound water, and O1 also acts as a hydrogen bond acceptor to unbound water. Both complexes are arranged in layers parallel to (101). Within and between the layers, these hydrogen bonds assemble the complexes and unbound species into a 3−D hydrogen-bonded network.

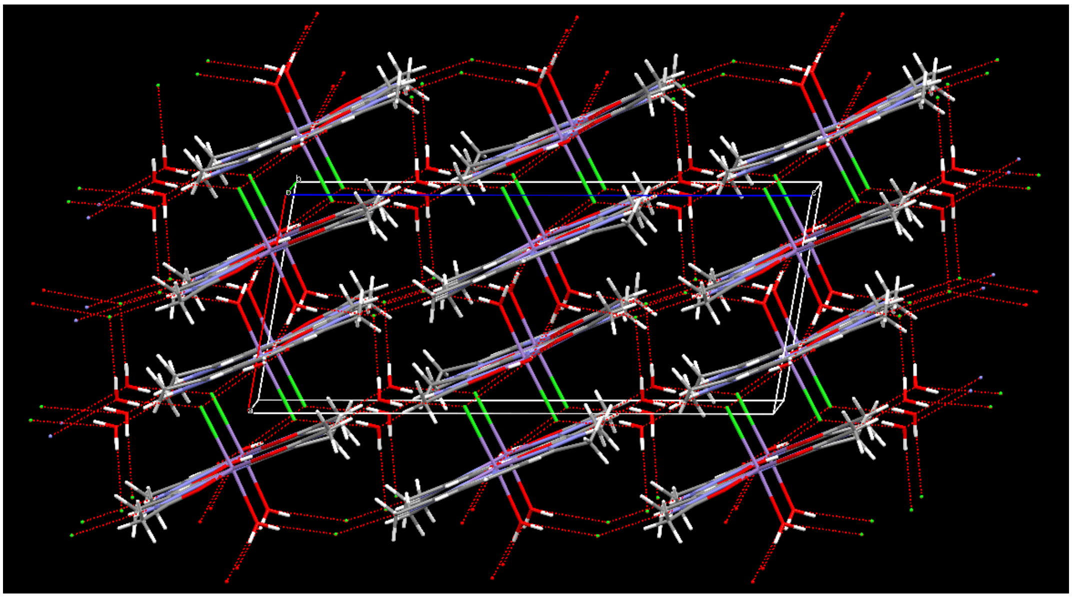

Figure S7. Crystal packing diagram of **MnL^{Me}**.

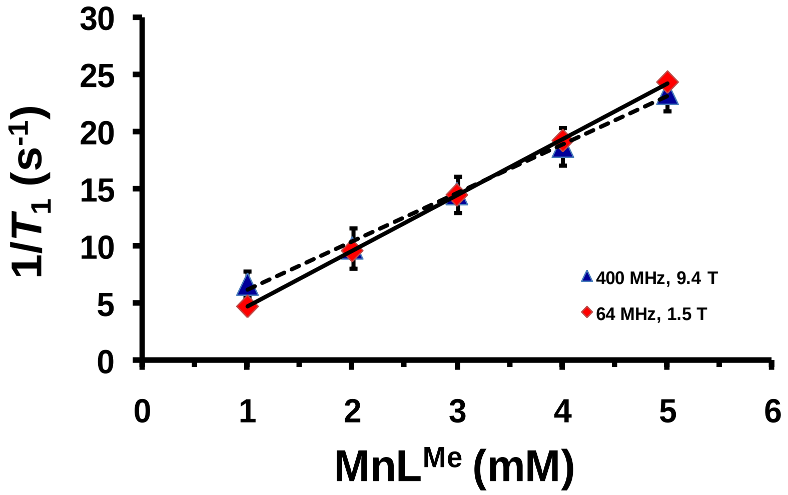

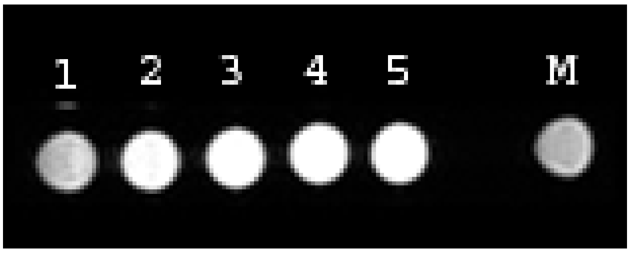

FigureS8. The plot of 1/T_1 vs. [**MnL^{Me}**] where the T_1 values determined at 9.4 T, 298 K and 1.5 T, 298 K (Top) and T_1-weighted MRI phantom images (1.5 T, 298 K) as a function of **MnL^{Me}** concentration (1 to 5 mM) and clinical standard Magnevist (M) (1 mM) (Bottom).

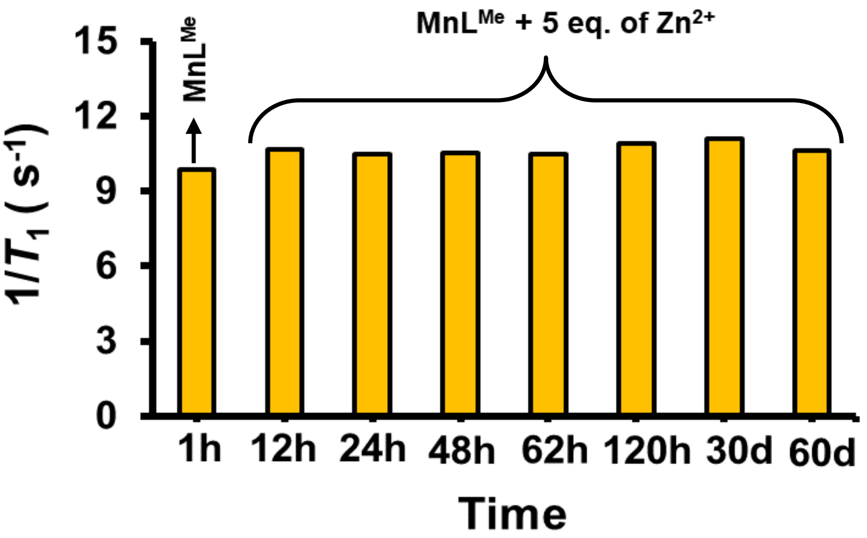

FigureS9. Transmetallation of 2 mM Mn^{2+} complex **MnL^{Me}** by 10 mM Zn^{2+} monitored by relaxation rate; R_1 ($1/T_1$, s^{-1}) change as a function of time in water, 400 MHz (9.4 T), 298K, (A: **MnL^{Me}** alone).

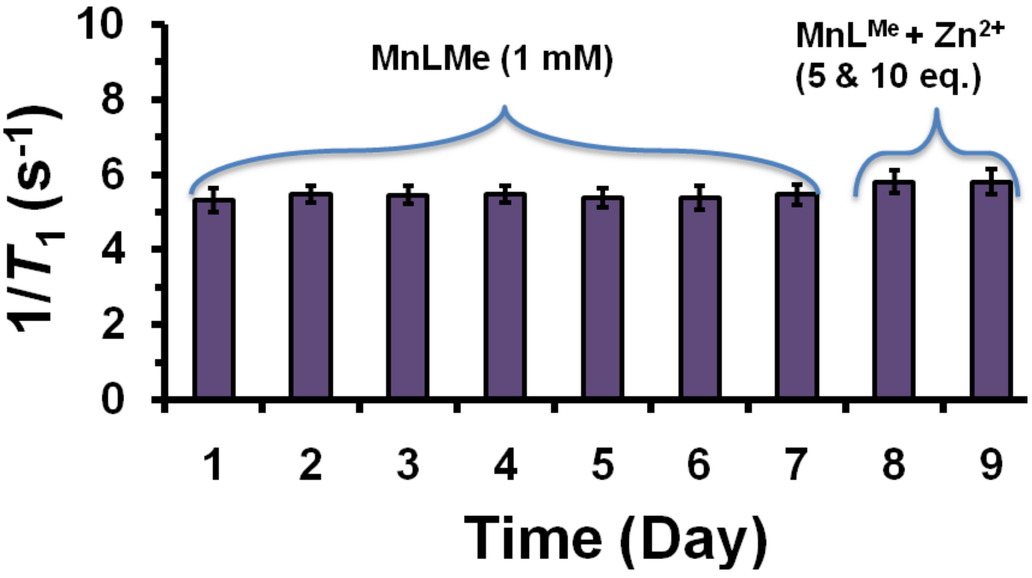

Figure S10. Time-dependent relaxivity and Zn^{2+} (5 and 10 mM) transmetallation of **MnL^{Me}** (1 mM) in water, 400 MHz (9.4 T), 298 K. (5 and 10 mM aqueous solutions of Zn^{2+} were added to the stock solution of **MnL^{Me}** (10 mM) and made up 5 and 10 equivalent solutions on day 8).

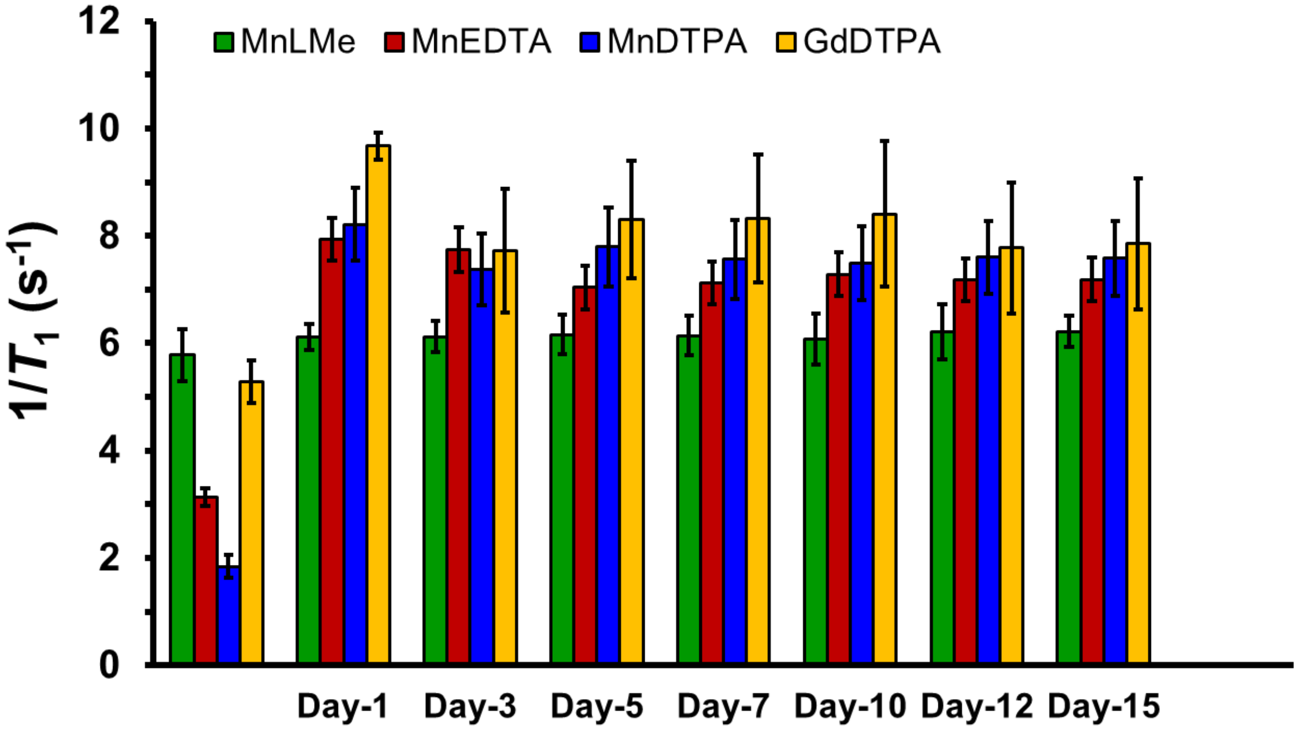

Figure S11. Time-dependent relaxivity and Zn^{2+} (25 mM) transmetallation of1 mM solutions of **MnL^{Me}**, $[Mn(EDTA)(H_2O)]^{2-}$, $[Mn(DTPA)]^{3-}$ and $[Gd(DTPA)]^{2-}$ in 50 mM MES (2-(N-morpholino)ethanesulfonic acid) buffer at pH 6.0, 400 MHz (9.4 T), 298 K.

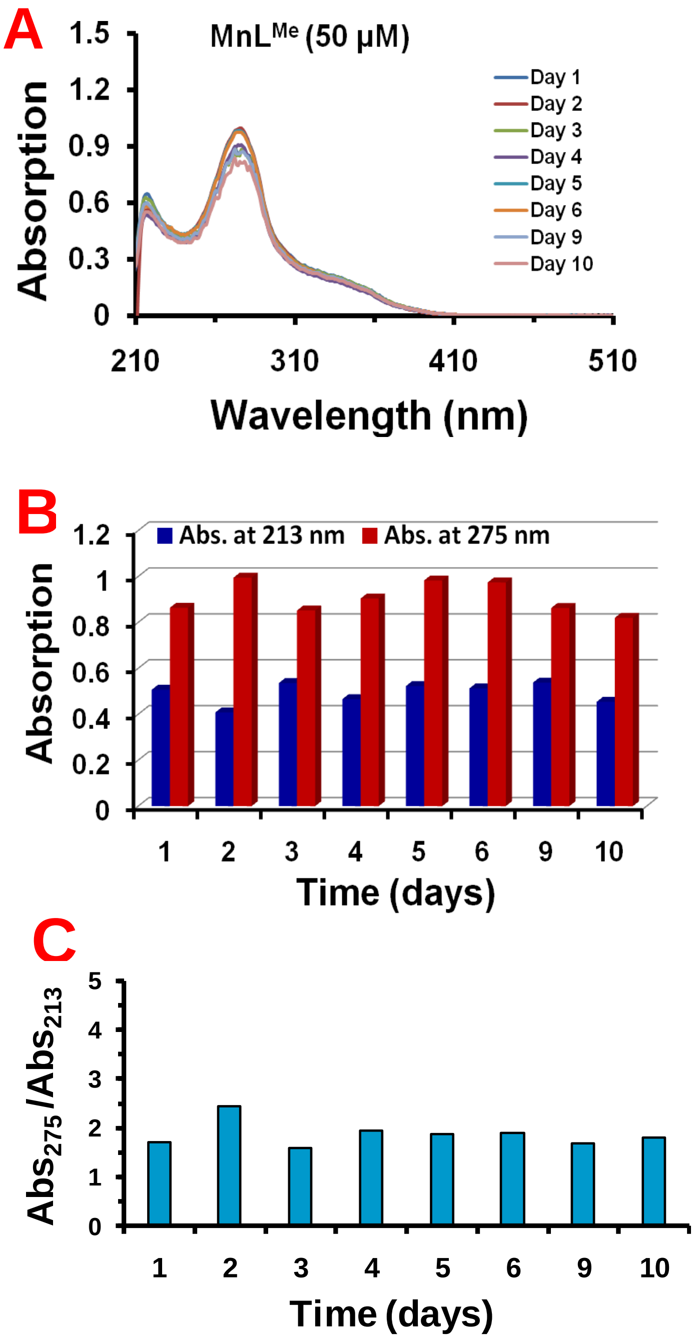

Figure S12. Chelating stability of **MnL^{Me}** was monitored by change in the absorption spectral intensity as a function of time. Absorption spectra of **MnL^{Me}** (50µM) obtained over 10 days (A), Change in the absorption intensities at 213 and 275 nm (B), and the ratio of Abs_{275}/Abs_{213} as the function of time (C). To reduce the errors during measurement, the ratio of absorption maxima λmax values was plotted with a function of time

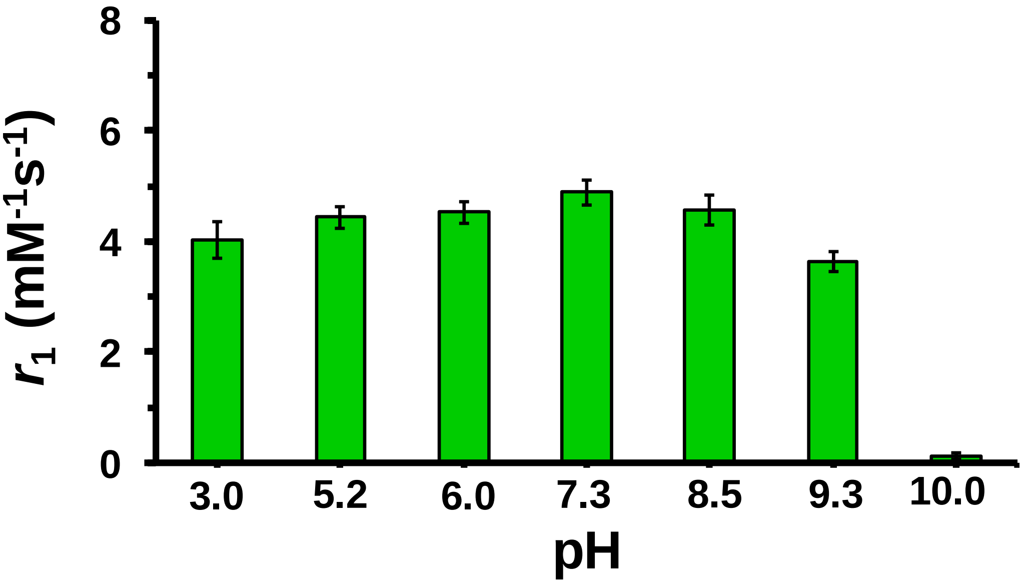

Figure S13. Change inrelaxivity of **MnL^{Me}**(1 mM)in 50 mMaqueous (phosphate/phosphoric acid (pH 3.0), MES (pH 5.2 and 6.0), HEPES (pH 7.3), Potassium phosphate/KOH (pH 8.5), NH_4Cl/NH_3 (pH 9.3 and 10.0) buffer solutions, 400 MHz (9.4 T), 25 °C.

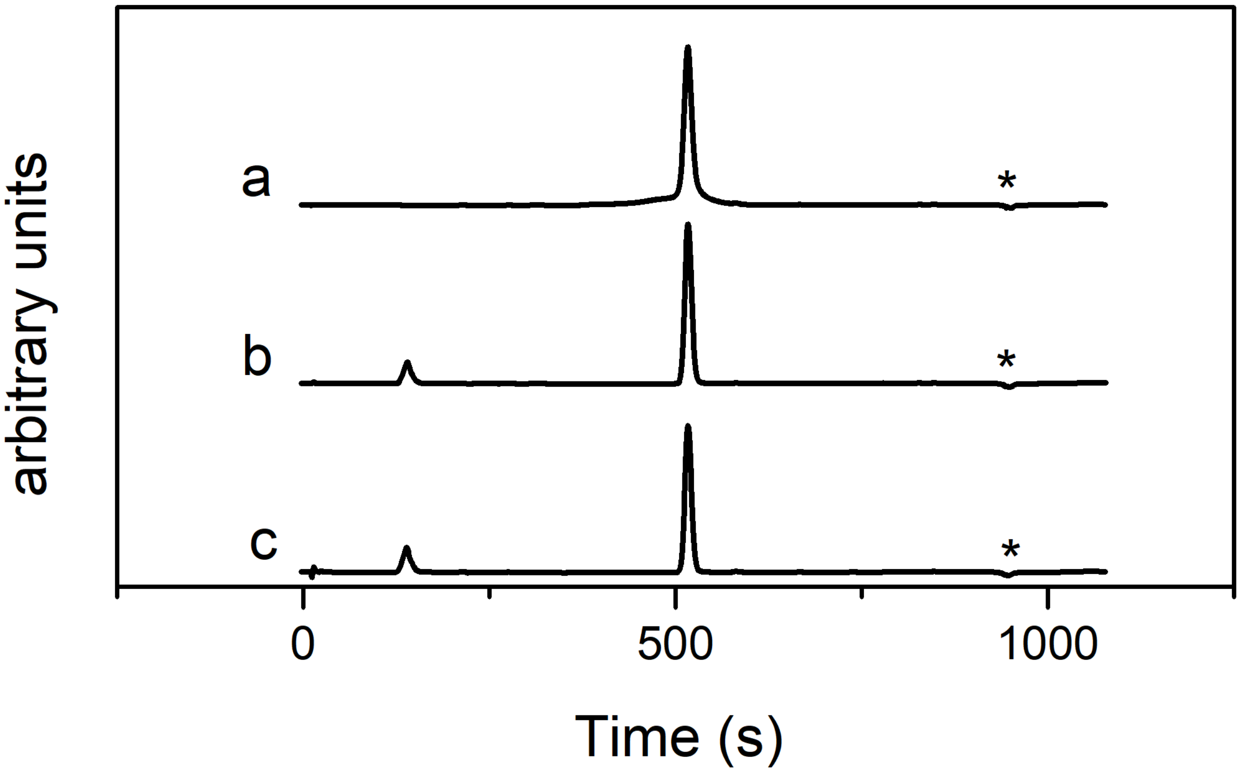

Figure S14. Transchelation of **MnL^{Me}**. No transchelation was observed in competition with 25 molar equivalents of DTPA (b), nor with 25 molar equivalents EDTA (c) in comparison to pure **MnL^{Me}** (a) Solutions were incubated for 30 mins with the competing ligands. The asterisk (*) marks a signal from the eluent system.

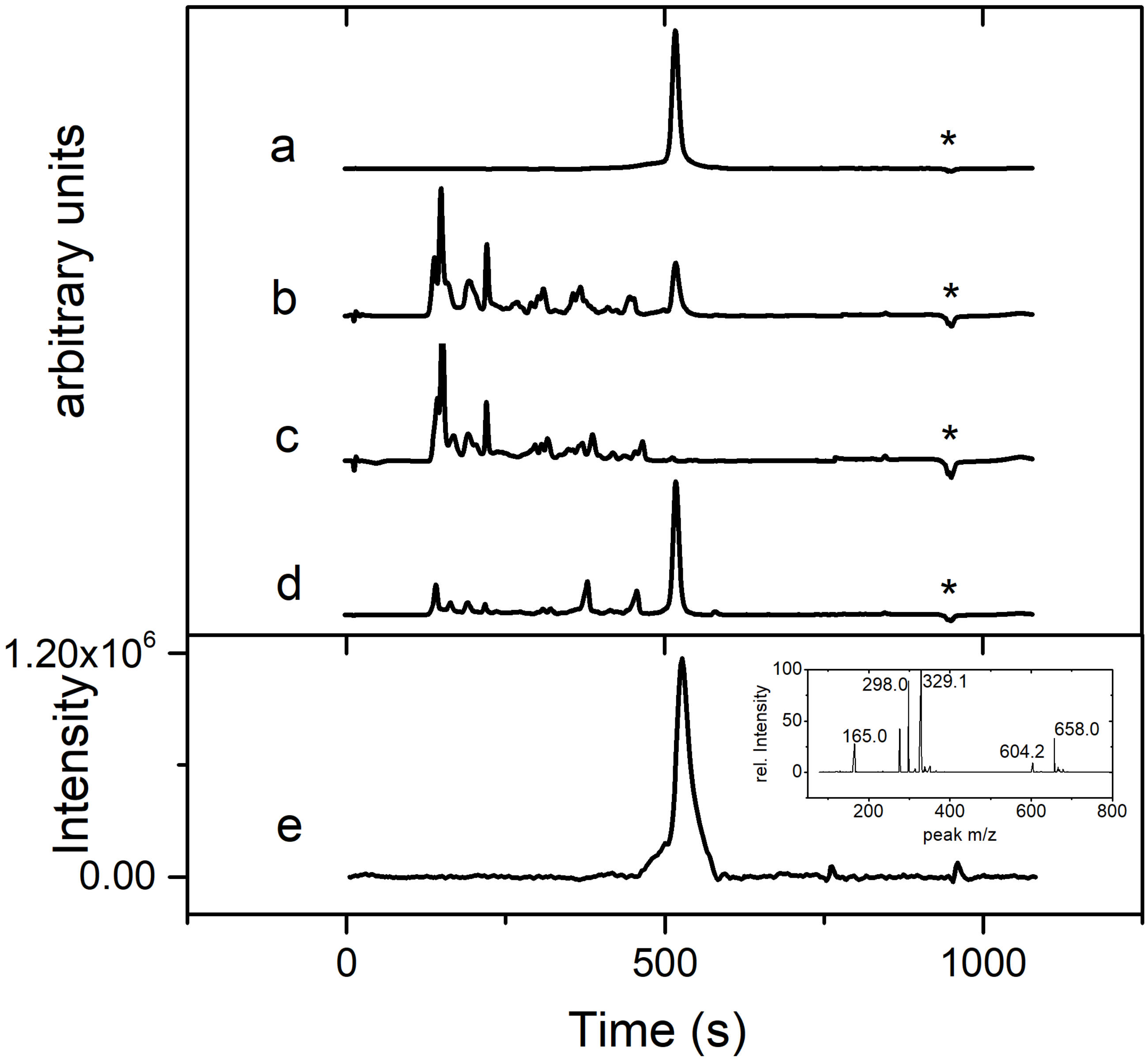

Figure S15. LC-MS of mouse urine samples. LC-MS analysis of pure **MnL^{Me}**(a) and a urine sample from a healthy mouse after intravenous administration of a clinical dose of **MnL^{Me}** (b). A control urine sample without prior **MnL^{Me}** administration (c) and a urine sample spiked with **MnL^{Me}** (d) on an Agilent LC-MS system (LC: 1200 series, MS: 6120 quadrupole). MS chromatogram (e) of the pure **MnL^{Me}** extracted at *m/z*+ 329.1 and the ESI-MS spectra at 520 sec (8.6 min). At 40 min post intravenous **MnL^{Me}** (0.1 mmol/kg bodyweight, $2.5x10^{-6}$ M) injection, 24 % of the contrast agent was excreted intact into the bladder. The asterisk (*) marks a signal from the eluent system.

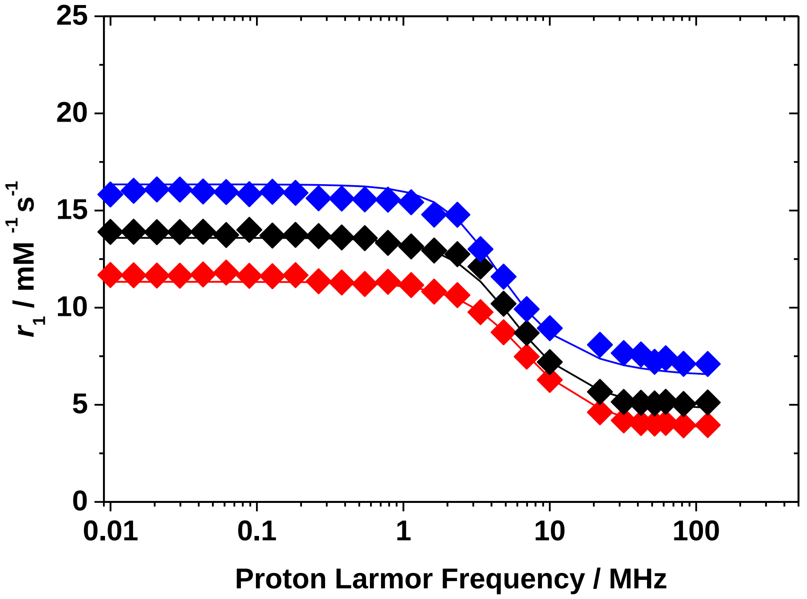

FigureS16. ^{1}H NMRD profiles of **MnL^{Me}** at pH 6 and three different temperatures (283 K (◆), 298 K (◆) and 310 K (◆). [Mn^{2+}] = 4.0 mM. The curves through the data points were calculated with the parameters in Table S11.

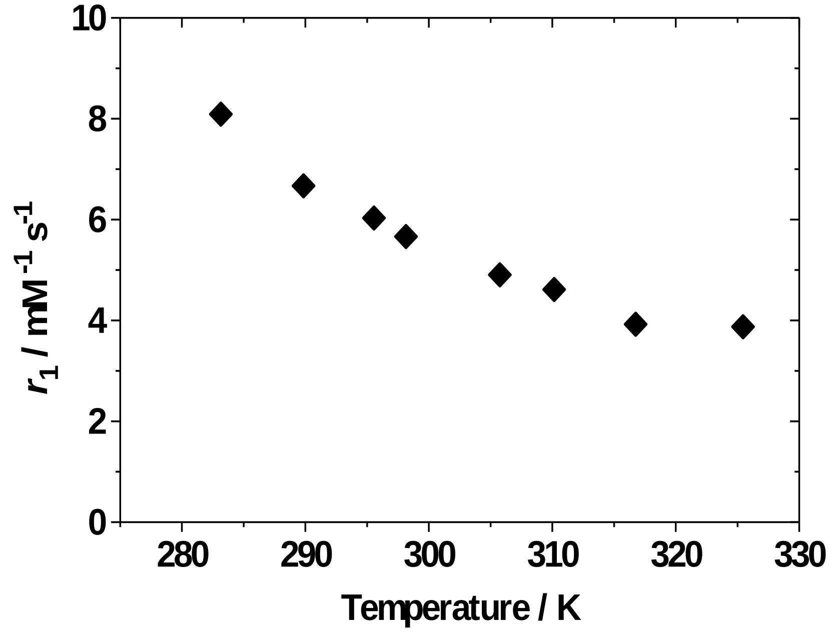

Figure S17. Temperature dependence of the proton relaxivity of **MnL^{Me}** (22 MHz).

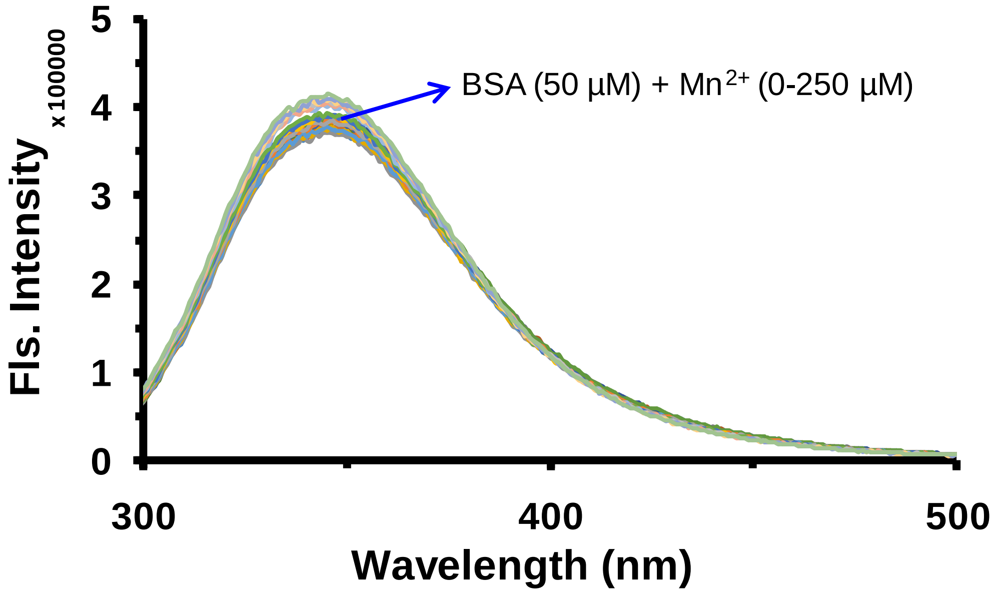

FigureS18. Fluorescence spectral profile of 50 µM BSA protein in 50 mM HEPES buffer (pH 7.4, 298 K) with increasing quantity of aq. $\mathbf{Mn^{2+}}$ solution (0–250 µM, λ_{ex} = 280 nm).

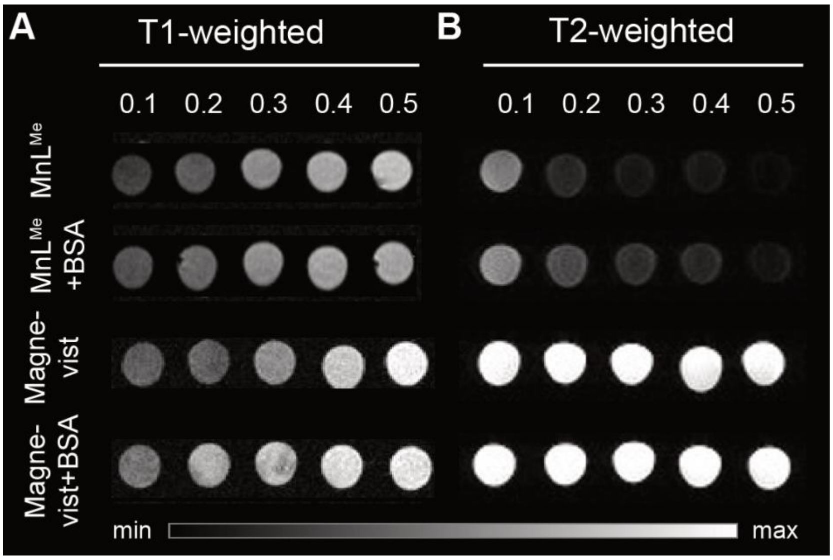

Figure S19. T_1 and T_2-weighted phantom imaging of MnL^{Me} and Magnevist. (A) T_1-weighted imaging of 0.1-0.5 mM **MnL^{Me}** without and with 0.6 mM BSA in 50 mM HEPES (first and second row, respectively) illustrates highly enhanced contrast enhancement in **MnL^{Me}** samples with BSA in comparison to **MnL^{Me}** samples without BSA. Compared to 0.1-0.5 mM Magnevist without and with 0.6 mM BSA in 50 mM HEPES (third and fourth row, respectively), overall contrast enhancement of **MnL^{Me}** samples is lower. Images were recorded at 7 T and 298 K with a flip angle of 10°. (B) T_2-weighted imaging of 0.1-0.5 mM **MnL^{Me}** without and with 0.6 mM BSA in 50 mM HEPES (first and second row, respectively) shows superior T_2 contrast modulation of **MnL^{Me}** in comparison to 0.1-0.5 mM Magnevist without and with 0.6 mM BSA in 50 mM HEPES (third and fourth row, respectively).

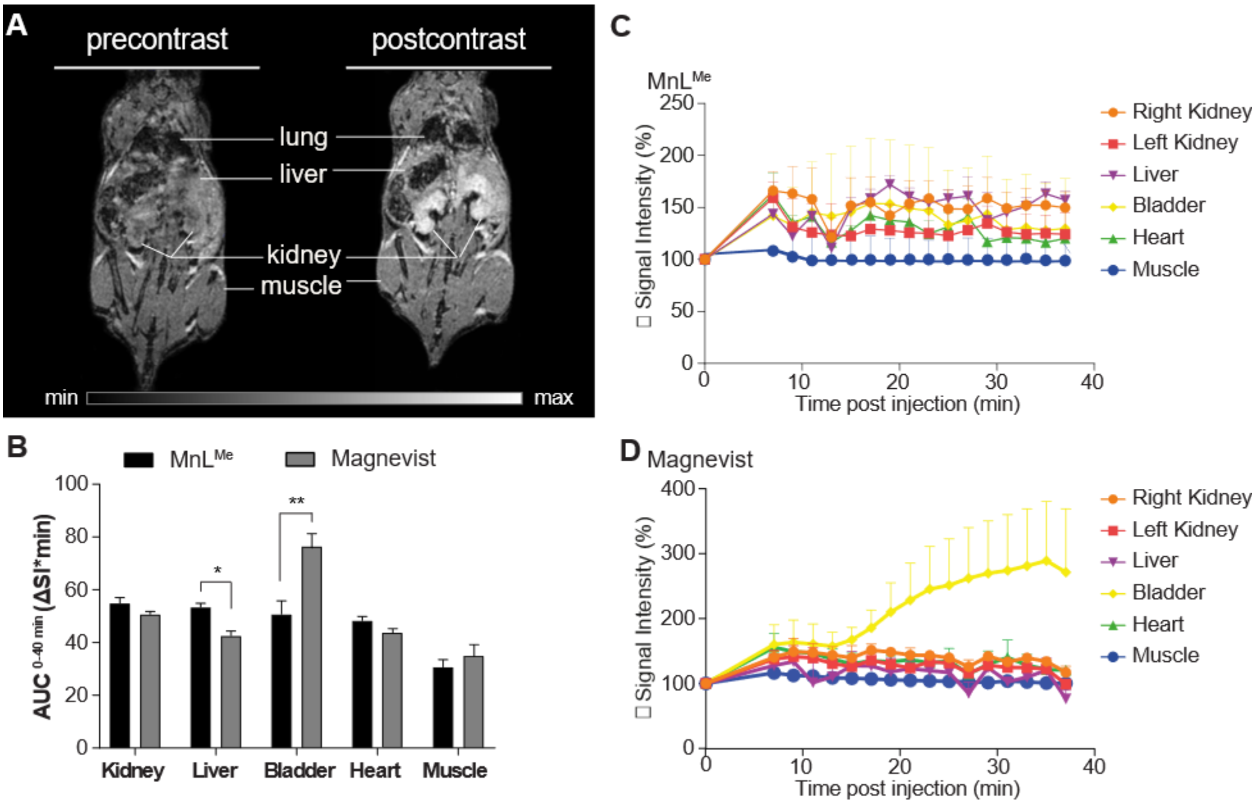

Figure S20: Biodistribution of Magnevist and **MnL^{Me}: (**A) Representative images of Magnevist®-induced contrast enhancement *in vivo*. (B) Total signal intensity over time of **MnL^{Me}** represented as the area under the curve (AUC$^{0\text{-}40\ min}$ (ΔSI*min)) was comparable to Magnevist in all analyzed organs (mean ± SEM, n = 3 for **MnL^{Me}**, n = 3 for Magnevist®). Percentage of signal intensity enhancement normalized to pre-injection over time for all organs of interest is given in (C) for **MnL^{Me}** and (D) for Magnevist®. Bars represent standard error of the mean; *p-value < 0.05, **p-value < 0.01.

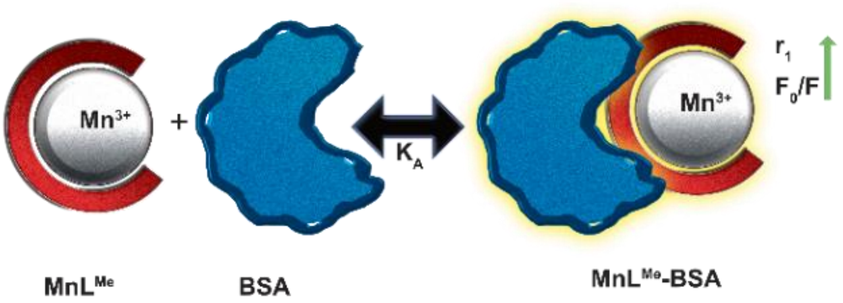

Scheme S1. MnL^{Me} binds with bovine serum albumin (BSA), resulting in an enhanced relaxivity due to the decreased molecular rotation.

Table S1. Crystallographic data for **MnL^{Me}** and**ZnL^{Me}**

**Parameters**	**MnL^{Me}**	**ZnL^{Me}**
Formula unit	$C_{13}H_{23}Cl_2MnN_5O_5$	$C_{13}H_{23}Cl_2N_5O_5Zn$
Formula weight	455.20	465.63
T (K)	150(2)	150(2)
Crystal system	Monoclinic	Monoclinic
Space group	P 21/c	P 21/c
a (Å)	8.0344(7)	7.9265(5)
b (Å)	13.1800(7)	13.2381(10)
c (Å)	19.0413(14)	18.7719(11)
β (°)	99.852(6)	99.377(5)
Volume (A^3)	1986.6(3)	1943.4(2)
Z	4	4
D_{calc} (Mg m^{-3}) and μ (mm^{-1})	1.522, 0.967	1.591, 1.573
F(000), Crystal size	940,	960, 0.150 x 0.110 x 0.070 mm^3
Theta range for data collection	1.888 to 26.371°	1.891 to 26.372°
Index ranges	-7 ≤ h ≤ 10, -14 ≤ k ≤ 16, -23 ≤ l ≤ 23	-6<=h<=9, -16<=k<=15, -23<=l<=23
Reflections collected	8383	9904
Independent reflections	4057	3911 [R(int) = 0.0987]
Completeness to theta = 98.3 %	26.371°	25.242°
Absorption correction	multi-scan	Semi-empirical from equivalents
Max. and min. transmission	0.901 and 0.910	0.942 and 0.918
Refinement method	Full-matrix least-squares on F^2	Full-matrix least-squares on F^2
Data / restraints / parameters	4027/9/269	3911 / 9 / 259
Goodness-of-fit on F^2	0.850	0.856
Final R indices [I>2sigma(I)]	R1 = 0.0322, wR2 = 0.0678	R1 = 0.0515, wR2 = 0.1088
R indices (all data)	R1 = 0.0686, WR2 = 0.0611	R1 = 0.1124, wR2 = 0.1248

Largest diff. peak and hole	0.287 and -0.277 e.Å^{-3}	0.529 and -0.415 e.Å^{-3}

$R_1 = \Sigma||F_o| - |F_c||/\Sigma|F_o|$. $wR_2 = [\Sigma[w(F_o^2 - F_c^2)^2]/ \Sigma[w(F_o^2)^2]]^{1/2}$

Table S2. Details of all of the unique classical hydrogen bonds in **MnL^{Me}**.

D−H	H...A	D...A	<(DHA)	
0.95	2.82	3.761(3)	171.2	C2−H2...Cl2_$i
0.95	2.79	3.516(3)	133.6	C4−H4...Cl1_$ii
0.98	2.94	3.898(3)	164.5	C7−H7B...Cl2_$i
0.98	2.69	3.592(3)	153.5	C9−H9B...Cl2_$iii
0.98	2.58	3.549(4)	168.7	C9−H9C...O2_$iv
0.98	2.61	3.405(4)	138.3	C11−H11C...O1W
0.98	2.49	3.349(4)	145.6	C13−H13B...O1W
0.82	2.00	2.788(3)	161.8	N5−H5...O1W
0.83	2.39	3.194(2)	163.3	N3−H3A...Cl2_$iii
0.80(3)	2.30(3)	3.0905(18)	168(3)	O3−H3B...Cl2
0.80(3)	1.86(3)	2.654(3)	173(3)	O3−H3C...O2W
0.80(2)	2.40(3)	3.153(2)	156(4)	O2W−H2A...Cl1_$v
0.79(2)	2.36(2)	3.124(2)	161(3)	O1W−H1B...Cl1_$vi
0.81(2)	2.10(2)	2.833(3)	151(3)	O2W−H2B...O1_$iv
0.80(2)	2.33(2)	3.131(3)	177(3)	O1W−H1A...Cl2_$vii

Symmetry equivalent atoms are generated by the following operators: i = −x+1, −y+1, −z+1, ii = −x, −y+1, −z+1, iii = x, −y+3/2, z−1/2, iv = −x+1, −y+2, −z+1, v = x+1, y, zvi = x, −y+3/2, z+1/2, vii = x−1, y, z

Table S3. Atomic coordinates (x 10^4) and equivalent isotropic displacement parameters (Å^2 x 10^3) for **MnL^{Me}**. U(eq) is defined as one third of the trace of the orthogonalized U^{ij} tensor.

	x	y	z	U(eq)
C(1)	3124(4)	5663(2)	4383(1)	24(1)
C(2)	3180(4)	4610(2)	4341(1)	30(1)

C(3)	2617(4)	4047(2)	4865(1)	36(1)
C(4)	2045(4)	4528(2)	5422(1)	32(1)
C(5)	2038(4)	5586(2)	5440(1)	26(1)
C(6)	3717(4)	6360(2)	3856(1)	24(1)
C(7)	4356(4)	5966(2)	3217(1)	37(1)
C(8)	3945(4)	9013(2)	3831(1)	23(1)
C(9)	4596(4)	9844(2)	3428(1)	32(1)
C(10)	1463(4)	6191(2)	6011(1)	26(1)
C(11)	1041(4)	5685(2)	6667(1)	40(1)
C(12)	1036(4)	8827(2)	6179(1)	27(1)
C(13)	471(4)	9591(2)	6670(1)	37(1)
N(1)	2551(3)	6130(1)	4921(1)	23(1)
N(4)	1410(3)	7146(2)	5892(1)	24(1)
N(5)	922(3)	7832(2)	6357(1)	28(1)
N(2)	3605(3)	7303(1)	4013(1)	23(1)
N(3)	4123(3)	8049(2)	3602(1)	24(1)
O(1)	3278(2)	9176(1)	4360(1)	26(1)
O(2)	1604(2)	9083(1)	5645(1)	27(1)
O(3)	4854(2)	7873(2)	5591(1)	31(1)
Mn(1)	2354(1)	7864(1)	4940(1)	21(1)
Cl(1)	-454(1)	7830(1)	4138(1)	30(1)
Cl(2)	5407(1)	6947(1)	7105(1)	37(1)
O(1W)	-784(3)	7562(2)	7502(1)	66(1)
O(2W)	7463(3)	8839(2)	5215(1)	46(1)

Table S4. Bond lengths [Å] and angles [°] for **MnL^{Me}**.

C(1)-N(1)	1.342(3)
C(1)-C(2)	1.392(3)
C(1)-C(6)	1.497(3)
C(2)-C(3)	1.380(4)
C(2)-H(2)	0.9500
C(3)-C(4)	1.381(4)
C(3)-H(3)	0.9500
C(4)-C(5)	1.394(4)
C(4)-H(4)	0.9500
C(5)-N(1)	1.341(3)
C(5)-C(10)	1.486(4)
C(6)-N(2)	1.284(3)

C(6)-C(7)	1.494(3)
C(7)-H(7A)	0.9800
C(7)-H(7B)	0.9800
C(7)-H(7C)	0.9800
C(8)-O(1)	1.238(3)
C(8)-N(3)	1.358(3)
C(8)-C(9)	1.483(3)
C(9)-H(9A)	0.9800
C(9)-H(9B)	0.9800
C(9)-H(9C)	0.9800
C(10)-N(4)	1.279(3)
C(10)-C(11)	1.504(3)
C(11)-H(11A)	0.9800
C(11)-H(11B)	0.9800
C(11)-H(11C)	0.9800
C(12)-O(2)	1.230(3)
C(12)-N(5)	1.362(3)
C(12)-C(13)	1.497(3)
C(13)-H(13A)	0.9800
C(13)-H(13B)	0.9800
C(13)-H(13C)	0.9800
N(1)-Mn(1)	2.2922(19)
N(4)-N(5)	1.369(3)
N(4)-Mn(1)	2.2872(18)
N(5)-H(5)	0.82(3)
N(2)-N(3)	1.365(3)
N(2)-Mn(1)	2.2979(19)
N(3)-H(3A)	0.83(3)
O(1)-Mn(1)	2.2446(16)
O(2)-Mn(1)	2.2418(16)
O(3)-Mn(1)	2.173(2)
O(3)-H(3B)	0.80(3)
O(3)-H(3C)	0.80(3)
Mn(1)-Cl(1)	2.4990(8)
O(1W)-H(1B)	0.79(2)
O(1W)-H(1A)	0.80(2)
O(2W)-H(2A)	0.80(2)
O(2W)-H(2B)	0.81(2)
N(1)-C(1)-C(2)	121.3(2)

N(1)-C(1)-C(6)	114.9(2)
C(2)-C(1)-C(6)	123.8(2)
C(3)-C(2)-C(1)	118.5(2)
C(3)-C(2)-H(2)	120.7
C(1)-C(2)-H(2)	120.7
C(2)-C(3)-C(4)	120.1(2)
C(2)-C(3)-H(3)	120.0
C(4)-C(3)-H(3)	120.0
C(3)-C(4)-C(5)	118.7(2)
C(3)-C(4)-H(4)	120.6
C(5)-C(4)-H(4)	120.6
N(1)-C(5)-C(4)	120.9(2)
N(1)-C(5)-C(10)	115.2(2)
C(4)-C(5)-C(10)	123.8(2)
N(2)-C(6)-C(7)	124.9(2)
N(2)-C(6)-C(1)	113.3(2)
C(7)-C(6)-C(1)	121.8(2)
C(6)-C(7)-H(7A)	109.5
C(6)-C(7)-H(7B)	109.5
H(7A)-C(7)-H(7B)	109.5
C(6)-C(7)-H(7C)	109.5
H(7A)-C(7)-H(7C)	109.5
H(7B)-C(7)-H(7C)	109.5
O(1)-C(8)-N(3)	120.5(2)
(1)-C(8)-C(9)	122.2(2)
N(3)-C(8)-C(9)	117.4(2)
C(8)-C(9)-H(9A)	109.5
C(8)-C(9)-H(9B)	109.5
H(9A)-C(9)-H(9B)	109.5
C(8)-C(9)-H(9C)	109.5
H(9A)-C(9)-H(9C)	109.5
H(9B)-C(9)-H(9C)	109.5
N(4)-C(10)-C(5)	113.8(2)
N(4)-C(10)-C(11)	125.3(2)
C(5)-C(10)-C(11)	120.9(2)
C(10)-C(11)-H(11A)	109.5
C(10)-C(11)-H(11B)	109.5
H(11A)-C(11)-H(11B)	109.5
C(10)-C(11)-H(11C)	109.5

H(11A)-C(11)-H(11C)	109.5
H(11B)-C(11)-H(11C)	109.5
O(2)-C(12)-N(5)	121.4(2)
O(2)-C(12)-C(13)	121.8(2)
N(5)-C(12)-C(13)	116.8(2)
C(12)-C(13)-H(13A)	109.5
C(12)-C(13)-H(13B)	109.5
H(13A)-C(13)-H(13B)	109.5
C(12)-C(13)-H(13C)	109.5
H(13A)-C(13)-H(13C)	109.5
H(13B)-C(13)-H(13C)	109.5
C(5)-N(1)-C(1)	120.4(2)
C(5)-N(1)-Mn(1)	119.42(16)
C(1)-N(1)-Mn(1)	120.15(15)
C(10)-N(4)-N(5)	122.63(19)
C(10)-N(4)-Mn(1)	122.89(16)
N(5)-N(4)-Mn(1)	114.24(14)
C(12)-N(5)-N(4)	115.78(19)
C(12)-N(5)-H(5)	122.1
C(6)-N(2)-N(3)	121.6(2)
C(6)-N(2)-Mn(1)	123.13(16)
N(3)-N(2)-Mn(1)	115.09(13)
C(8)-N(3)-N(2)	115.56(18)
C(8)-N(3)-H(3A)	122.2
N(2)-N(3)-H(3A)	122.2
C(8)-O(1)-Mn(1)	119.48(15)
C(12)-O(2)-Mn(1)	118.27(16)
Mn(1)-O(3)-H(3B)	128(3)
Mn(1)-O(3)-H(3C)	127(2)
H(3B)-O(3)-H(3C)	103(3)
O(3)-Mn(1)-O(2)	88.32(7)
O(3)-Mn(1)-O(1)	85.73(7)
O(2)-Mn(1)-O(1)	83.58(6)
O(3)-Mn(1)-N(4)	87.07(7)
O(2)-Mn(1)-N(4)	70.24(7)
O(1)-Mn(1)-N(4)	153.01(7)
O(3)-Mn(1)-N(1)	87.43(8)
O(2)-Mn(1)-N(1)	138.62(7)
O(1)-Mn(1)-N(1)	136.98(7)

N(4)-Mn(1)-N(1)	68.44(7)
O(3)-Mn(1)-N(2)	87.59(7)
O(2)-Mn(1)-N(2)	152.72(7)
O(1)-Mn(1)-N(2)	69.23(6)
N(4)-Mn(1)-N(2)	136.37(7)
N(1)-Mn(1)-N(2)	68.08(7)
O(3)-Mn(1)-Cl(1)	177.17(5)
O(2)-Mn(1)-Cl(1)	94.09(5)
O(1)-Mn(1)-Cl(1)	93.07(5)
N(4)-Mn(1)-Cl(1)	95.13(5)
N(1)-Mn(1)-Cl(1)	91.72(6)
N(2)-Mn(1)-Cl(1)	89.59(6)
H(1B)-O(1W)-H(1A)	106(3)
H(2A)-O(2W)-H(2B)	106(3)

Symmetry transformations used to generate equivalent atoms:

Table S5. Anisotropic displacement parameters ($Å^2$x 10^3) for **MnL^{Me}**. The anisotropic displacement factor exponent takes the form: $-2\pi^2$[$h^2a^{*2}U^{11}$ + ... + 2 h k a* b* U^{12}]

	U^{11}	U^{22}	U^{33}	U^{23}	U^{13}	U^{12}
C(1)	25(2)	19(1)	27(1)	-2(1)	-3(1)	-1(1)
C(2)	29(2)	21(1)	38(1)	-4(1)	-1(1)	1(1)
C(3)	31(2)	20(1)	53(2)	2(1)	-1(1)	-2(1)
C(4)	26(2)	24(1)	42(1)	8(1)	0(1)	-5(1)
C(5)	21(2)	23(1)	32(1)	7(1)	1(1)	-3(1)
C(6)	24(2)	23(1)	26(1)	-3(1)	2(1)	3(1)
C(7)	52(2)	30(2)	30(1)	-7(1)	12(1)	7(1)
C(8)	26(2)	23(1)	21(1)	0(1)	6(1)	-3(1)
C(9)	47(2)	24(1)	29(1)	-1(1)	16(1)	-11(1)
C(10)	22(2)	28(1)	29(1)	10(1)	3(1)	-5(1)
C(11)	41(2)	44(2)	37(1)	18(1)	11(1)	-5(2)
C(12)	26(2)	33(2)	22(1)	0(1)	6(1)	2(1)
C(13)	43(2)	43(2)	29(1)	-2(1)	15(1)	10(2)
N(1)	23(1)	19(1)	27(1)	0(1)	4(1)	-2(1)
N(4)	21(1)	29(1)	23(1)	2(1)	5(1)	-1(1)
N(5)	30(1)	35(1)	21(1)	6(1)	12(1)	3(1)
N(2)	26(1)	20(1)	23(1)	0(1)	6(1)	-3(1)

N(3)	32(1)	23(1)	21(1)	-1(1)	12(1)	-3(1)
O(1)	36(1)	20(1)	24(1)	-2(1)	14(1)	-2(1)
O(2)	34(1)	25(1)	25(1)	0(1)	13(1)	1(1)
O(3)	30(1)	36(1)	29(1)	4(1)	7(1)	-7(1)
Mn(1)	26(1)	18(1)	20(1)	0(1)	9(1)	-2(1)
Cl(1)	31(1)	32(1)	26(1)	3(1)	4(1)	-1(1)
Cl(2)	47(1)	43(1)	23(1)	2(1)	12(1)	-3(1)
O(1W)	45(2)	122(2)	35(1)	18(1)	18(1)	-6(2)
O(2W)	45(2)	24(1)	78(2)	-4(1)	35(1)	-2(1)

Table S6. Hydrogen coordinates (x 10^4) and isotropic displacement parameters (Å^{2}x 10^3) for **MnL^{Me}**.

	x	y	z	U(eq)
H(2)	3596	4286	3961	36
H(3)	2623	3327	4842	43
H(4)	1663	4146	5787	38
H(7A)	3700	6266	2785	56
H(7B)	4238	5226	3196	56
H(7C)	5549	6148	3248	56
H(9A)	3695	10340	3280	48
H(9B)	4976	9564	3006	48
H(9C)	5546	10177	3732	48
H(11A)	1984	5772	7063	60
H(11B)	844	4960	6573	60
H(11C)	22	5994	6792	60
H(13A)	1439	10001	6889	56
H(13B)	-6	9239	7043	56
H(13C)	-391	10032	6400	56
H(5)	581(12)	7654(6)	6722(12)	33
H(3A)	4521(13)	7922(4)	3237(12)	29
H(3B)	5120(40)	7690(30)	5995(14)	69
H(3C)	5680(40)	8160(30)	5513(17)	69
H(2A)	7790(50)	8690(20)	4852(15)	69
H(1B)	-540(40)	7580(30)	7923(12)	69
H(2B)	7410(50)	9449(17)	5213(17)	69
H(1A)	-1760(30)	7410(30)	7414(17)	69

Table S7. Atomic coordinates (x 10^4) and equivalent isotropic displacement parameters (Å^{2}x 10^3) for **ZnL^{Me}**. U(eq) is defined as one third of the trace of the orthogonalized U^{ij} tensor.

	x	y	z	U(eq)
C(1)	3123(7)	5670(4)	4412(2)	24(1)
C(2)	3138(8)	4628(4)	4377(3)	30(1)
C(3)	2562(9)	4079(4)	4916(3)	36(1)
C(4)	2001(7)	4572(4)	5481(3)	30(1)
C(5)	2027(7)	5625(4)	5491(2)	26(1)
C(6)	3686(8)	6350(4)	3870(2)	27(1)
C(7)	4299(9)	5957(4)	3204(3)	36(2)
C(8)	3908(7)	8983(4)	3856(2)	25(1)
C(9)	4538(8)	9839(4)	3439(3)	33(1)
C(10)	1459(7)	6254(4)	6061(2)	28(1)
C(11)	976(9)	5792(5)	6726(3)	37(2)
C(12)	1105(7)	8889(4)	6167(2)	27(1)
C(13)	532(8)	9677(4)	6643(3)	35(1)
N(1)	2563(6)	6145(3)	4958(2)	24(1)
N(4)	1449(6)	7197(3)	5924(2)	25(1)
N(5)	969(6)	7906(3)	6379(2)	27(1)
N(2)	3609(6)	7292(3)	4031(2)	24(1)
N(3)	4119(5)	8044(3)	3614(2)	24(1)
O(1)	3297(5)	9135(3)	4412(2)	30(1)
O(2)	1704(5)	9095(3)	5619(2)	30(1)
O(3)	4889(5)	7835(3)	5597(2)	33(1)
Zn(1)	2428(1)	7811(1)	4971(1)	23(1)
Cl(1)	-311(2)	7817(1)	4205(1)	32(1)
Cl(2)	5539(2)	6928(1)	7120(1)	39(1)
O(1W)	-725(6)	7779(5)	7583(2)	56(1)
O(2W)	7465(7)	8873(3)	5231(2)	49(1)

Table S8. Bond lengths [Å] and angles [°] for **ZnL^{Me}**

C(1)-N(1)	1.338(7)
C(1)-C(2)	1.382(7)
C(1)-C(6)	1.480(7)

C(2)-C(3)	1.382(8)
C(2)-H(2)	0.9500
C(3)-C(4)	1.379(8)
C(3)-H(3)	0.9500
C(4)-C(5)	1.394(8)
C(4)-H(4)	0.9500
C(5)-N(1)	1.339(6)
C(5)-C(10)	1.484(8)
C(6)-N(2)	1.287(6)
C(6)-C(7)	1.506(7)
C(7)-H(7A)	0.9800
C(7)-H(7B)	0.9800
C(7)-H(7C)	0.9800
C(8)-O(1)	1.236(6)
C(8)-N(3)	1.344(7)
C(8)-C(9)	1.508(7)
C(9)-H(9A)	0.9800
C(9)-H(9B)	0.9800
C(9)-H(9C)	0.9800
C(10)-N(4)	1.274(7)
C(10)-C(11)	1.494(7)
C(11)-H(11A)	0.9800
C(11)-H(11B)	0.9800
C(11)-H(11C)	0.9800
C(12)-O(2)	1.231(6)
C(12)-N(5)	1.370(7)
C(12)-C(13)	1.491(8)
C(13)-H(13A)	0.9800
C(13)-H(13B)	0.9800
C(13)-H(13C)	0.9800
N(1)-Zn(1)	2.208(4)
N(4)-N(5)	1.365(6)
N(4)-Zn(1)	2.218(4)
N(5)-H(5)	0.8800
N(2)-N(3)	1.367(6)
N(2)-Zn(1)	2.237(4)
N(3)-H(3A)	0.8800
O(1)-Zn(1)	2.210(4)
O(2)-Zn(1)	2.220(4)

O(3)-Zn(1)	2.107(4)
O(3)-H(3B)	0.771(19)
O(3)-H(3C)	0.78(2)
Zn(1)-Cl(1)	2.4006(15)
O(1W)-H(1B)	0.74(4)
O(1W)-H(1A)	0.73(4)
O(2W)-H(2A)	0.80(4)
O(2W)-H(2B)	0.77(4)
N(1)-C(1)-C(2)	120.8(5)
N(1)-C(1)-C(6)	114.6(4)
C(2)-C(1)-C(6)	124.6(5)
C(3)-C(2)-C(1)	118.9(5)
C(3)-C(2)-H(2)	120.5
C(1)-C(2)-H(2)	120.5
C(4)-C(3)-C(2)	120.1(5)
C(4)-C(3)-H(3)	120.0
C(2)-C(3)-H(3)	120.0
C(3)-C(4)-C(5)	118.5(5)
C(3)-C(4)-H(4)	120.8
C(5)-C(4)-H(4)	120.8
N(1)-C(5)-C(4)	120.7(5)
N(1)-C(5)-C(10)	114.9(5)
C(4)-C(5)-C(10)	124.4(5)
N(2)-C(6)-C(1)	113.4(4)
N(2)-C(6)-C(7)	124.2(5)
C(1)-C(6)-C(7)	122.4(5)
C(6)-C(7)-H(7A)	109.5
C(6)-C(7)-H(7B)	109.5
H(7A)-C(7)-H(7B)	109.5
C(6)-C(7)-H(7C)	109.5
H(7A)-C(7)-H(7C)	109.5
H(7B)-C(7)-H(7C)	109.5
O(1)-C(8)-N(3)	121.6(5)
O(1)-C(8)-C(9)	121.7(5)
N(3)-C(8)-C(9)	116.7(4)
C(8)-C(9)-H(9A)	109.5
C(8)-C(9)-H(9B)	109.5
H(9A)-C(9)-H(9B)	109.5
C(8)-C(9)-H(9C)	109.5

H(9A)-C(9)-H(9C)	109.5
H(9B)-C(9)-H(9C)	109.5
N(4)-C(10)-C(5)	113.4(4)
N(4)-C(10)-C(11)	125.2(5)
C(5)-C(10)-C(11)	121.4(5)
C(10)-C(11)-H(11A)	109.5
C(10)-C(11)-H(11B)	109.5
H(11A)-C(11)-H(11B)	109.5
C(10)-C(11)-H(11C)	109.5
H(11A)-C(11)-H(11C)	109.5
H(11B)-C(11)-H(11C)	109.5
O(2)-C(12)-N(5)	120.9(5)
O(2)-C(12)-C(13)	122.8(5)
N(5)-C(12)-C(13)	116.3(4)
C(12)-C(13)-H(13A)	109.5
C(12)-C(13)-H(13B)	109.5
H(13A)-C(13)-H(13B)	109.5
C(12)-C(13)-H(13C)	109.5
H(13A)-C(13)-H(13C)	109.5
H(13B)-C(13)-H(13C)	109.5
C(1)-N(1)-C(5)	121.0(4)
C(1)-N(1)-Zn(1)	120.0(3)
C(5)-N(1)-Zn(1)	119.0(3)
C(10)-N(4)-N(5)	122.8(4)
C(10)-N(4)-Zn(1)	122.0(4)
N(5)-N(4)-Zn(1)	115.0(3)
N(4)-N(5)-C(12)	115.3(4)
N(4)-N(5)-H(5)	122.3
C(12)-N(5)-H(5)	122.3
C(6)-N(2)-N(3)	122.8(4)
C(6)-N(2)-Zn(1)	121.6(3)
N(3)-N(2)-Zn(1)	115.4(3)
C(8)-N(3)-N(2)	114.5(4)
C(8)-N(3)-H(3A)	122.7
N(2)-N(3)-H(3A)	122.7
C(8)-O(1)-Zn(1)	117.9(3)
C(12)-O(2)-Zn(1)	117.2(4)
Zn(1)-O(3)-H(3B)	119(5)
Zn(1)-O(3)-H(3C)	127(5)

H(3B)-O(3)-H(3C)	110(4)
O(3)-Zn(1)-N(1)	88.78(17)
O(3)-Zn(1)-O(1)	85.46(14)
N(1)-Zn(1)-O(1)	140.13(16)
O(3)-Zn(1)-N(4)	88.24(14)
N(1)-Zn(1)-N(4)	70.51(16)
O(1)-Zn(1)-N(4)	148.34(14)
O(3)-Zn(1)-O(2)	88.93(15)
N(1)-Zn(1)-O(2)	142.00(15)
O(1)-Zn(1)-O(2)	77.38(12)
N(4)-Zn(1)-O(2)	71.51(14)
O(3)-Zn(1)-N(2)	88.62(15)
N(1)-Zn(1)-N(2)	69.97(16)
O(1)-Zn(1)-N(2)	70.48(14)
N(4)-Zn(1)-N(2)	140.40(14)
O(2)-Zn(1)-N(2)	147.86(14)
O(3)-Zn(1)-Cl(1)	176.97(11)
N(1)-Zn(1)-Cl(1)	92.13(13)
O(1)-Zn(1)-Cl(1)	91.99(10)
N(4)-Zn(1)-Cl(1)	94.80(11)
O(2)-Zn(1)-Cl(1)	92.12(11)
N(2)-Zn(1)-Cl(1)	88.97(11)
H(1B)-O(1W)-H(1A)	107(4)
H(2A)-O(2W)-H(2B)	97(3)

Symmetry transformations used to generate equivalent atoms:

Table S9. Anisotropic displacement parameters ($Å^2$x 10^3)for **ZnL^{Me}**. The anisotropic displacement factor exponent takes the form: $-2\pi^2$[$h^2a^{*2}U^{11}$ + ... + 2 h k a* b* U^{12}]

	U^{11}	U^{22}	U^{33}	U^{23}	U^{13}	U^{12}
C(1)	22(3)	25(3)	26(2)	-4(2)	2(2)	2(2)
C(2)	37(4)	20(3)	33(3)	-2(2)	3(2)	4(2)
C(3)	38(3)	17(2)	49(3)	3(2)	-2(3)	-1(3)
C(4)	29(3)	24(3)	36(3)	4(2)	5(2)	-6(2)
C(5)	26(3)	24(3)	28(2)	3(2)	4(2)	-2(2)
C(6)	37(4)	25(3)	21(2)	-2(2)	6(2)	7(3)
C(7)	52(4)	27(3)	32(3)	-5(2)	13(3)	5(3)

C(8)	31(3)	25(3)	22(2)	1(2)	13(2)	-1(3)
C(9)	50(4)	24(3)	29(2)	-2(2)	19(2)	-12(3)
C(10)	27(3)	31(3)	27(2)	6(2)	5(2)	-3(3)
C(11)	44(4)	34(3)	36(3)	13(2)	13(3)	-7(3)
C(12)	28(3)	30(3)	25(2)	-4(2)	9(2)	1(3)
C(13)	43(4)	35(3)	28(2)	-2(2)	11(2)	4(3)
N(1)	32(2)	15(2)	25(2)	2(2)	8(2)	2(2)
N(4)	30(3)	24(3)	24(2)	-3(2)	10(2)	-1(2)
N(5)	32(3)	27(3)	25(2)	2(2)	13(2)	3(2)
N(2)	27(2)	19(2)	29(2)	1(2)	10(2)	-3(2)
N(3)	33(3)	20(2)	23(2)	1(2)	15(2)	-3(2)
O(1)	42(3)	21(2)	29(2)	-3(1)	14(2)	-4(2)
O(2)	38(3)	25(2)	30(2)	0(2)	16(2)	-2(2)
O(3)	38(2)	29(2)	35(2)	4(2)	19(2)	-7(2)
Zn(1)	31(1)	18(1)	24(1)	1(1)	11(1)	-2(1)
Cl(1)	37(1)	29(1)	32(1)	2(1)	8(1)	-1(1)
Cl(2)	52(1)	39(1)	30(1)	2(1)	14(1)	-1(1)
O(1W)	44(3)	85(4)	41(2)	19(3)	18(2)	-14(3)
O(2W)	53(3)	21(2)	79(3)	0(2)	33(3)	-4(2)

Table S10. Hydrogen coordinates (x 10^4) and isotropic displacement parameters (Å^{2}x 10^3) for **ZnL^{Me}**.

	x	y	z	U(eq)
H(2)	3539	4294	3989	36
H(3)	2552	3362	4897	43
H(4)	1605	4202	5855	36
H(7A)	3549	6208	2773	54
H(7B)	4277	5217	3206	54
H(7C)	5470	6191	3199	54
H(9A)	3588	10298	3269	50
H(9B)	4995	9566	3024	50
H(9C)	5441	10208	3753	50
H(11A)	1803	5995	7148	56
H(11B)	978	5054	6681	56
H(11C)	-168	6022	6784	56
H(13A)	1514	10088	6855	52
H(13B)	33	9350	7028	52
H(13C)	-328	10109	6357	52

H(5)	598	7744	6781	32
H(3A)	4550	7923	3219	29
H(3B)	4990(80)	7720(60)	6004(14)	73
H(3C)	5700(60)	8110(60)	5510(30)	73
H(2A)	7590(110)	8790(50)	4820(20)	73
H(1B)	-540(90)	7870(60)	7980(20)	73
H(2B)	7460(110)	9450(30)	5230(30)	73
H(1A)	-1590(60)	7570(60)	7500(30)	73

Table S11. Parameters from the analysis of ^{1}H NMRD data.

Parameters	$[MnL^{Me}]^{2+}$ (MW = 366 g/mol)	[Mn(dpama)]Ref-[7] (MW = 390 g/mol)	$[Mn(EDTA)]^{2-}$ Ref-[8] (MW = 361 g/mol)	[b]$[Mn(H_2O)_6]^{2+}$Ref-[7] (MW = 163 g/mol)
$^{298}r_1$ 20 MHz / mM^{-1} s^{-1}	5.7	5.3	3.3	7.7
Δ^2 / 10^{19} s^{-2}	0.81±0.12	2.38	6.9	0.6
$\tau_V^{298\,K}$/ ps	48.0±1.1	39.2	27.9	10.0
E_V / kJ mol^{-1}	12.4±1.5	1.0	1.0	14.6
$^{298}\tau_M$/ ns	191±9	3.3	2.1	35.5
ΔH_M / kJ mol^{-1}	29.7±1.2	28.1	33.5	45.6
$^{298}\tau_R$/ ps	52.0±0.1	47.8	57	30
E_R / kJ mol^{-1}	16.0[a]	25.3	21.8	16.7
$A_O/\hbar$/10^6 rad s^{-1}	28±1	45.8	40.5	34.6
q	2[a]	2	1	6
r / Å	2.74[a]	2.74	2.83	2.83
a / Å	3.6[a]	3.6	3.6	3.6
^{298}D / 10^5 cm^2 s^{-1}	2.30[a]	2.24	2.31	2.30
E_D / kJ mol^{-1}	16.0±0.5	17.3	18.9	29.7

[a]fixed in the fitting procedure.

Table S12. Parameters from the analysis of ^{1}H NMRD and ^{17}O NMR data of **MnL^{Me} /** BSA.

Parameters	MnL^{Me} / BSA[b]
Δ^2 / 10^{19} s^{-2}	2.1±0.3

τ_V / ps	3.7±0.6
τ_M / ns	378±11
τ_{RG} / ps	50000[a]
τ_{RL} / ps	408±45
S^2	0.28±0.01
$A_O/\hbar$ /10^6 rad s^{-1}	30±2
q	1
r / Å	2.74[a]
a / Å	3.6[a]
D / 10^5 cm^2 s^{-1}	2.30[a]

[a]fixed in the fitting procedure

References

[1] X-AREA v 1.64, STOE & Cie GmbH, Darmstadt, **2012**.

[2] G. M. Sheldrick, *Acta Cryst*. **2015**, *A71*, 3–8.

[3] G. M. Sheldrick, *Acta Cryst*. **2015**, *C71*, 3–8.

[4] J. A. Peters,C. F. G. C.Geraldes, *Inorganics***2018**, *6*, 116.

[5] (a) D. F. Evans, *J. Chem. Soc*. **1959**, 2003–2005. (b) D. F. Evans, G. V. Fazakerley, R. F. Phillips, *J. Chem. Soc. (A)***1971***,* 1931–1934. (c) D. M. Corsi, C. P.-Iglesias, H. van Bekkum, J. A. Peters, Magn. Reson. Chem. 2001, 39, 723–726.

[6] E.M. Gale, J. Zhu, P. Caravan, *J. Am. Chem. Soc.*, **2013**, *135*, 18600−18608.

[7] A. Forgacs, M. Regueiro-Figueroa, J. L. Barriada, D. Esteban-Gómez, A. de Blas, T. Rodríguez-Blas, M. Botta, C. Platas-Iglesias, *Inorg. Chem*. **2015**, *54*, 9576−9587.

[8] A. Forgács, R. Pujales- Paradela, M. Regueiro-Figueroa, L. Valencia, D. Esteban-Gómez, M. Botta, C. Platas-Iglesias, *Dalton Trans*. **2017**, *46*, 1546−1558.

Author Contributions

S.A. synthesised and characterized the complexes **MnL^Me^** and **ZnL^Me^**, and carried out the relaxometry, transmetallation, pH-dependent relaxivity, PRE and BSA binding experiments (Figures 3CD, S1-S5, S8-S13 and S18). T. R. P. collected the single crystal X-ray data and obtained the crystal structures (Figures 1, S6 and S7). F.C. and M.B. recorded and analyzed the ^{1}H-NMRD profiles, BSA relaxometric titration and ^{17}O-NMR data (Figures 3AB, S16-S17). L.K. acquired the T_1-weighted phantom images (1-5 mM) from 1.5 T clinical MRI (Figure S8). TWP perform LCMS transchelation experiments. S. H. L. H and A. F.M. performed and analysed the transmetallation kinetics, *in vivo* MRI and T_1-weighted phantom imaging studies (Figures 2-4, S14, S15, S19 and S20). While all authors contributed to the text, G. J. S. and AFM coordinated the project. G.J.S. supervised and conceptualised the scientific work.
